# Risk factors for urosepsis following ureteroscopic lithotripsy: a systematic review and meta-analysis

**DOI:** 10.3389/fsurg.2025.1603311

**Published:** 2025-06-19

**Authors:** Lifei Dai, Junlian Xiang, Xiaoli Liu, Xiaoyan Wen, Lin Tan, Jiali Zhang

**Affiliations:** ^1^ School of Basic Medical Sciences & School of Nursing, Chengdu University, Chengdu, China; ^2^ Department of Urology, Deyang People’s Hospital, Deyang, China; ^3^ Department of Proctology, Deyang People’s Hospital, Deyang, China; ^4^ Department of Urology, The Affiliated Hospital of Chengdu University, Chengdu, China

**Keywords:** lithotripsy, sepsis, risk factors, disease prevention and control, meta-analysis

## Abstract

**Background:**

Ureteroscopic lithotripsy (URSL) is the preferred treatment for urinary tract stones, with urosepsis being its most severe postoperative complication. Although previous studies have investigated risk factors for urosepsis after URSL, significant variations exist in reported risk factors and their associated odds ratios (*OR*), leading to inconsistent findings across studies. This systematic review and meta-analysis investigated the risk factors for urosepsis after URSL, aiming to establish a scientific foundation for early clinical identification and to reduce the incidence and mortality of this complication.

**Methods:**

Case-control and cohort studies on factors influencing urosepsis after URSL were systematically retrieved from major public medical databases, including PubMed, Web of Science, Embase, Cochrane Library, China National Knowledge Infrastructure (CNKI), Wanfang Data, and Chinese Science and Technology Journal Database, up to January 31, 2025. Two researchers independently conducted literature screening, data extraction, quality assessment, and meta-analysis using Stata versions 15.1 and 18.0.

**Results:**

A total of 26 studies were included in this analysis, comprising 12,394 patients, of whom 861 patients developed urosepsis. The influencing factors for urosepsis included stone size[OR = 3.10, 95% CI (1.20,8.00), *P* = 0.002], number of stones [OR = 7.59, 95% confidence interval (CI): 3.82, 15.08; *P* < 0.001], history of urinary tract infection (OR = 5.96, 95% CI: 4.12, 8.60; *P* < 0.001), positive urine culture (OR = 4.95, 95% CI: 3.90, 6.28; *P* < 0.001), positive urinary nitrite (OR = 7.68, 95% CI: 1.03, 52.27; *P* = 0.047], C-reactive protein (OR = 4.3, 95% CI: 1.06, 17.49; *P* = 0.042), diabetes (OR = 3.60, 95% CI: 3.11, 4.16; *P* < 0.001), operation time (OR = 1.09, 95% CI: 1.07, 1.11; *P* < 0.001), and stent placement (OR = 3.71, 95% CI: 1.94, 7.09; *P* < 0.001].

**Conclusion:**

Urosepsis following URSL is associated with a high mortality rate and significantly threatens patient safety and quality of life. Early identification of the factors influencing urosepsis is crucial to reduce its incidence and improve patient outcomes.

**Systematic Review Registration:**

PROSPERO CRD42025641787.

## 1 Introduction

The global prevalence of urinary stones ranges from 1% to 20% (1), and ureteroscopic lithotripsy (URSL) is the preferred treatment for upper urinary tract stones due to its minimally invasive nature, quick recovery, and high success rate. For stones ≤2 cm, the clearance rate reaches 76%–100% (2–4). However, URSL is associated with a 9%–25% complication rate, including ureteral injury, bleeding, infection, and urosepsis. Urosepsis is the most serious complication, which can progress to septic shock without timely intervention. It may cause multiple organ dysfunction, affecting the brain, heart, and kidneys, with a mortality rate of 30%–40% (5).

Although multiple studies have examined risk factors of urosepsis after URSL, variations in reported factors and odds ratios (OR) have resulted in inconsistent conclusions. This systematic review and meta-analysis evaluated the risk factors for urosepsis following URSL and synthesized current evidence to support the development of early identification and intervention strategies. The findings aim to reduce both the incidence and mortality of postoperative urosepsis.

## 2 Materials and methods

This systematic review and meta-analysis was conducted following the Preferred Reporting Items for Systematic Reviews and Meta-Analyses (PRISMA) statement (6).

### 2.1 Search strategy

Two independent investigators searched PubMed, Web of Science, Embase, Cochrane Library, China National Knowledge Infrastructure (CNKI), Wanfang Data, and Viper Information Products databases. The search was conducted using a combination of subject terms and free words. English search terms: “Ureteroscopy/Lithotripsy/Ureteroscopy stone surgery/Ureteroscopic stone removal/Flexible ureteroscopy/Ureterolithotripsy/Ureteroscopic surgery/Ureteroscopic treatment,” “urosepsis/Urinary sepsis/ Septic shock/Urinary tract infection sepsis/Sepsis,” and “Risk factors/Influencing factors/Relevant factors/Associate factors/Predictive factors.” Additionally, references of the retrieved articles were manually reviewed to identify further relevant studies. The search covered the period from the inception of each database to January 31, 2025.

### 2.2 Inclusion and exclusion criteria

Inclusion criteria were as follows:

• Subjects: Patients undergoing URSL
• Type of study: case-control study or cohort study
• Study: Factors influencing or predictors of urosepsis after URSL
• Outcome metrics: OR and 95% CI for factors influencing urosepsis after URSL
• Diagnostic criteria: Urosepsis diagnosed within 30 days after URSL (7), with concurrent urinary tract infection (UTI) and systemic inflammatory response syndrome (SIRS) (8). SIRS was diagnosed based on ≥2 objective clinical parameters: (a) core hypothermia (<36°C) or hyperthermia (>38°C); (b) sustained tachycardia (>90 bpm); (c) tachypnea (>20 respirations/min) or documented hypocapnia [PaCO₂ < 32 mmHg (4.3 kPa)]; and (d) either leukocytopenia (<4 × 10⁹/L) or leukocytosis (>12 × 10⁹/L).

Exclusion criteria were as follows: duplicate publications, reviews, conference abstracts, or studies without full-text access; reports with incorrect, incomplete, or inaccessible data; and articles not in Chinese or English.

### 2.3 Data extraction

Two researchers skilled in meta-analysis methods independently removed duplicates using EndNote 20 and then screened the titles and abstracts of the remaining documents to eliminate those that did not meet the eligibility criteria. They reviewed the full text to determine the final inclusion of the selected reports. The screening results were cross-checked, and discrepancies were resolved by discussion; a third researcher adjudicated unresolved disagreements. Information about the relevant literature was extracted, including: first author, year of publication, country, type of study, sample size, number of patients with urosepsis, influencing factors, OR, and 95% confidence (CI).

### 2.4 Quality assessment of literature

The quality of bias in case-control and cohort studies was assessed using the Newcastle-Ottawa Scale (NOS) (9). This scale evaluates literature quality across three dimensions, comprising a total of eight entries: four entries for participant selection, one for group comparability, and three for outcome assessment. While each standard entry is scored on a 1-point scale, the comparability item allows for a maximum of 2 points, resulting in a total possible score of 9 points across all domains. Studies were categorized as low (0–4 points), medium (5–7 points), and high quality (>7 points). Two researchers independently evaluated the studies, and any differences in scoring were resolved through discussion, with the help of a third researcher.

### 2.5 Statistical analysis

Stata 18.0 was used for meta-analysis, subgroup analysis, and sensitivity analysis, while Egger's test was performed using Stata 15.1. All data were obtained directly from the original studies without any processing or transformation. The statistical results were presented as OR with 95% CI. Heterogeneity was evaluated using Cochran's *Q* test and *I*^2^ statistics. A fixed-effects model was applied when *I*^2^ < 50% and *P* > 0.10; otherwise, a random-effects model was used when *I*^2^ ≥ 50% and *P* ≤ 0.10. Subgroup analyses and sensitivity analyses were conducted to explore potential sources of heterogeneity. Funnel plots and Egger's test were used to assess publication bias when there were at least 10 studies, and the trim-and-fill method was applied when bias was detected. Statistical significance was set at *P* < 0.05.

## 3 Results

### 3.1 Literature search

**Figure 1:**
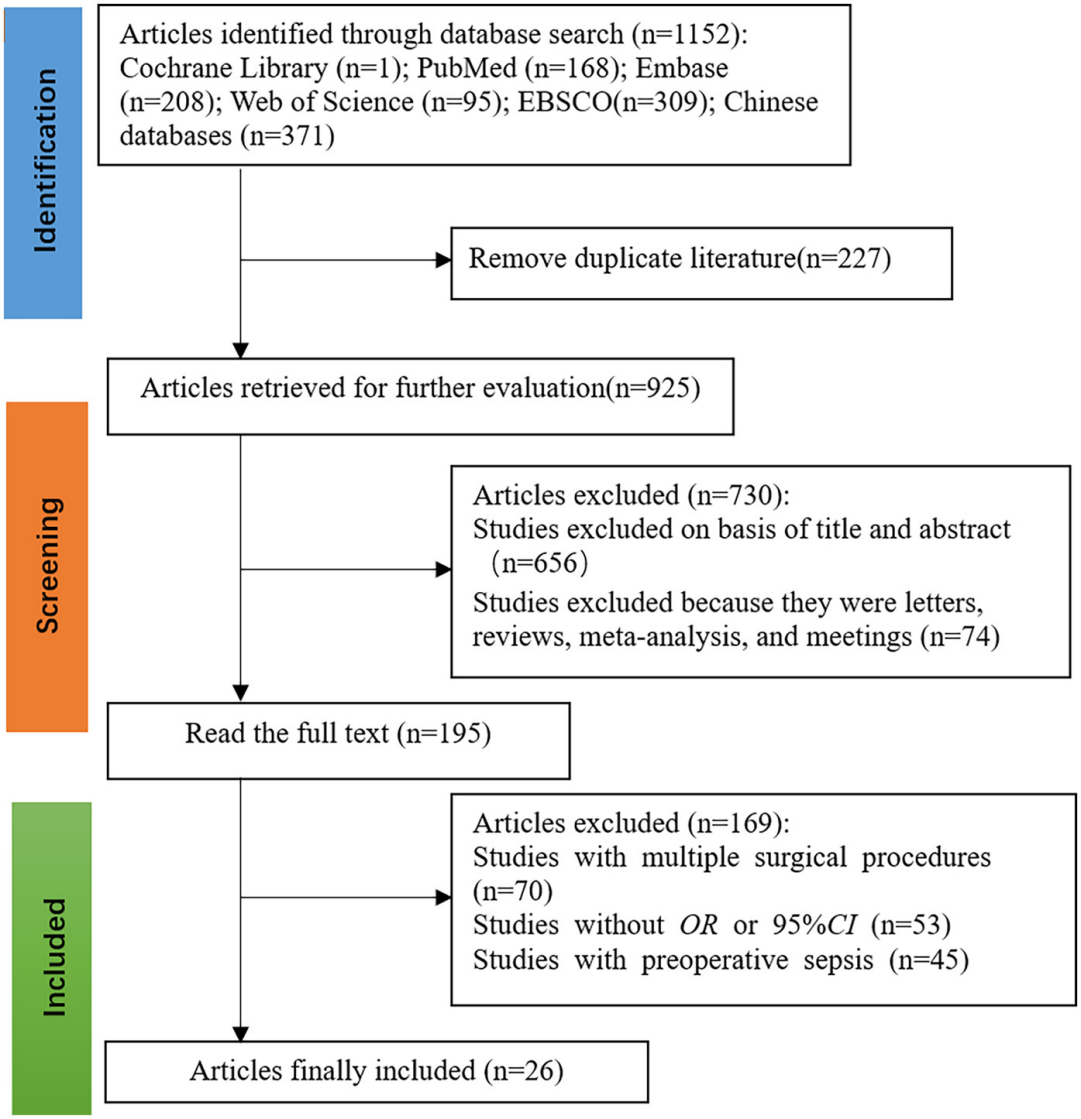
Flowchart of literature selection.

The initial search identified 1,152 potentially relevant articles. After a stepwise screening process, 26 studies (10–27) met the inclusion criteria, consisting of 16 articles published in English and 10 published in Chinese. The combined study population included 12,394 patients, of whom 861 cases were diagnosed with urosepsis. The literature screening process and results are illustrated in Figure 1.

### 3.2 Characteristics and quality of the included studies

The analysis identified 36 risk factors across all included studies, with 13 factors reported in at least two studies. Based on the NOS quality assessment, 13 studies were rated medium quality and 13 high quality; none of the articles were of low quality. The characteristics of the included studies and the results of the quality assessment are shown in Table 1.

**Table 1:** Characteristics and quality of included studies.

Author	Year	Area	Study type	Same size US cases		Influencing factors	NOS score
Amihay et al. (26)	2017	Israel	Cohort study	601	36	1, 2, 3	8
Blackmur et al. (20)	2016	England	Cohort study	426	34	2, 4	8
Can et al. (21)	2022	China	Cohort study	236	17	6, 7	8
Changgeng et al. (23)	2019	China	Case-Control	390	45	8, 9, 10	7
Chunyo et al. (27)	2022	China	Cohort study	830	32	5, 8, 11, 12, 13	8
Devos et al. (25)	2024	Belgium	Cohort study	400	6	5	8
Dingjun et al. (11)	2019	China	Case-Control	452	45	4, 8, 9, 27	7
Jarry et al. (22)	2022	France	Cohort study	282	27	14, 15	7
Jianxuan et al. (46)	2022	China	Cohort study	412	49	4, 16	7
Jincheng et al. (14)	2022	China	Case-Control	400	30	1, 9, 27, 34	7
Jonathan et al. (47)	2017	America	Cohort study	345	15	14	8
Junkai et al. (42)	2023	China	Case-Control	314	20	4, 16, 17, 18	7
Junlin et al. (48)	2020	China	Cohort study	759	43	4, 19	8
Krystian et al. (7)	2024	Poland	Cohort study	231	11	4	8
Leibo et al. (24)	2024	China	Cohort study	759	32	4, 20, 21, 22, 23	8
Linbin et al. (16)	2024	China	Case-Control	139	13	8, 14, 27, 32, 33	7
Maxime et al. (49)	2024	France	Cohort study	432	18	24	8
Mengjuan et al. (18)	2021	China	Case-Control	508	25	8, 14	7
Miaomiao et al. (28)	2023	China	Cohort study	1,060	29	8, 9, 14, 20, 25	8
Shimin et al. (13)	2022	China	Case-Control	400	90	6, 7, 25, 27, 28, 29	7
Suqiong et al. (17)	2024	China	Cohort study	600	50	1, 4, 8, 9, 27, 35	8
Tao Bai et al. (50)	2019	China	Cohort study	1,421	12	4, 8	8
Wenwei et al. (10)	2024	China	Case-Control	428	42	4, 7, 26	7
Xiaobo et al. (12)	2020	China	Case-Control	252	29	1, 4, 8, 9, 27	7
Xinhui et al. (15)	2023	China	Case-Control	120	60	1, 4, 8, 9, 27	7
Yongjie et al. (19)	2023	China	Case-Control	197	51	5, 8, 18, 30, 31	6

US: urosepsis; NOS: Newcastle-Ottawa Scale.

1. Gender; 2. Stent placement; 3. Stent indwelling time; 4. Positive urine culture; 5. Age; 6. Procalcitonin; 7. C-reactive protein; 8. Operation time; 9. Diabetes; 10. Irrigation pressure; 11. Stone location; 12. Hydronephrosis; 13. qSOFA or SOFA score; 14. History of urinary tract infection; 15. Neurological disease; 16. Albumin level; 17. Degree of albumin change; 18. White blood cell count; 19. Albumin-globulin ratio; 20. Positive urine nitrite; 21. Urine white blood cell count; 22. Neutrophil-lymphocyte ratio; 23. Residual stone; 24. Stone composition; 25. Number of stones; 26. Heparin-binding protein level; 27. Stone size; 28. Serum creatinine; 29. Blood urea nitrogen; 30. Platelet level; 31. History of urological surgery; 32. Antibiotic use; 33. Fever; 34. Stone obstruction; 35. Intraoperative irrigation fluid volume.

### 3.3 Meta-analysis

Meta-analysis was conducted for factors reported in at least two studies, including two demographic factors (gender, age), two stone-related factors (stone size, stone number), six infection-related factors [history of UTI, positive urine culture, positive urine nitrite, leukocyte count, C-reactive protein (CRP), procalcitonin], two surgical factors (operation time, stent placement), and one comorbidity-related factor (diabetes mellitus).

Stone size, stone number, history of UTI, positive urine culture, positive urine nitrite, CRP levels, operation time, stent placement, and diabetes mellitus were significantly associated with urosepsis following URSL (*P* < 0.05). However, no significant associations were found for gender, age, leukocyte count, or procalcitonin levels (*P* > 0.05), as shown in Table 2.

**Table 2:** Meta-analysis of risk factors for urosepsis after ureteroscopic lithotripsy.

Factors	Number of studies	Heterogeneity test		Effect model	Pooled effect	
		*I*^2^ (%)	*P* value		OR (95% CI)	*P* value
Gender	5	81	<0.001	Random	1.85 (0.75, 4.52)	0.18
Age	3	73.4	0.023	Random	1.09 (0.98, 1.2)	0.123
Stone size	7	92.9	<0.001	Random	3.10 (1.20, 8.00)	0.02
Number of stones	2	32.9	0.222	Fixed	7.59 (3.82, 15.08)	<0.001
History of urinary tract infection	5	80.7	<0.001	Random	7.90 (3.18, 19.64)	<0.001
Positive urine culture	12	29.8	0.154	Fixed	4.95 (3.90, 6.28)	<0.001
Positive urine nitrite	2	90.6	<0.001	Random	7.68 (1.03, 52.27)	0.047
White blood cell count	2	87.8	0.004	Random	1.12 (0.42, 3.0)	0.823
C-reactive protein	3	94.1	<0.001	Random	4.3 (1.06, 17.49)	0.042
Procalcitonin	2	95.6	<0.001	Random	9.34 (0.1, 878.49)	0.335
Operation time	11	88.9	<0.001	Random	1.52 (1.30, 1.77)	<0.001
Stent placement	2	0	0.758	Fixed	3.71 (1.94, 7.09)	<0.001
Diabetes	7	8.1	0.366	Fixed	3.60 (3.11, 4.16)	<0.001

### 3.4 Descriptive analysis

A total of 22 factors were reported in single studies, including: stone-related factors (stone location, residual stones, stone composition, and stone obstruction); procedure-related factors (stent retention time, irrigation pressure, intraoperative fluid volume, and antibiotic use); laboratory markers (albumin levels, albumin variability, albumin-globulin ratio, urinary leukocyte count, neutrophil-lymphocyte ratio, blood urea nitrogen, platelet count, heparin-binding protein level, and serum creatinine); underlying diseases (neurological disorders and history of urological surgery); and other factors (qSOFA/SOFA score, fever, and hydronephrosis). The clinical significance of these factors requires cautious interpretation due to limited evidence from single studies.

### 3.5 Subgroup and sensitivity analyses

For factors with *I*^2^ > 50%, subgroup analyses were conducted by sample size, study design, and number of urosepsis cases to identify potential sources of heterogeneity. When subgroup analysis was not feasible or significant heterogeneity persisted after subgroup analysis, sensitivity analysis was performed by sequentially excluding individual studies to assess changes in the combined *OR* values and *P* values, helping to identify possible sources of heterogeneity.

**Figure 2:**
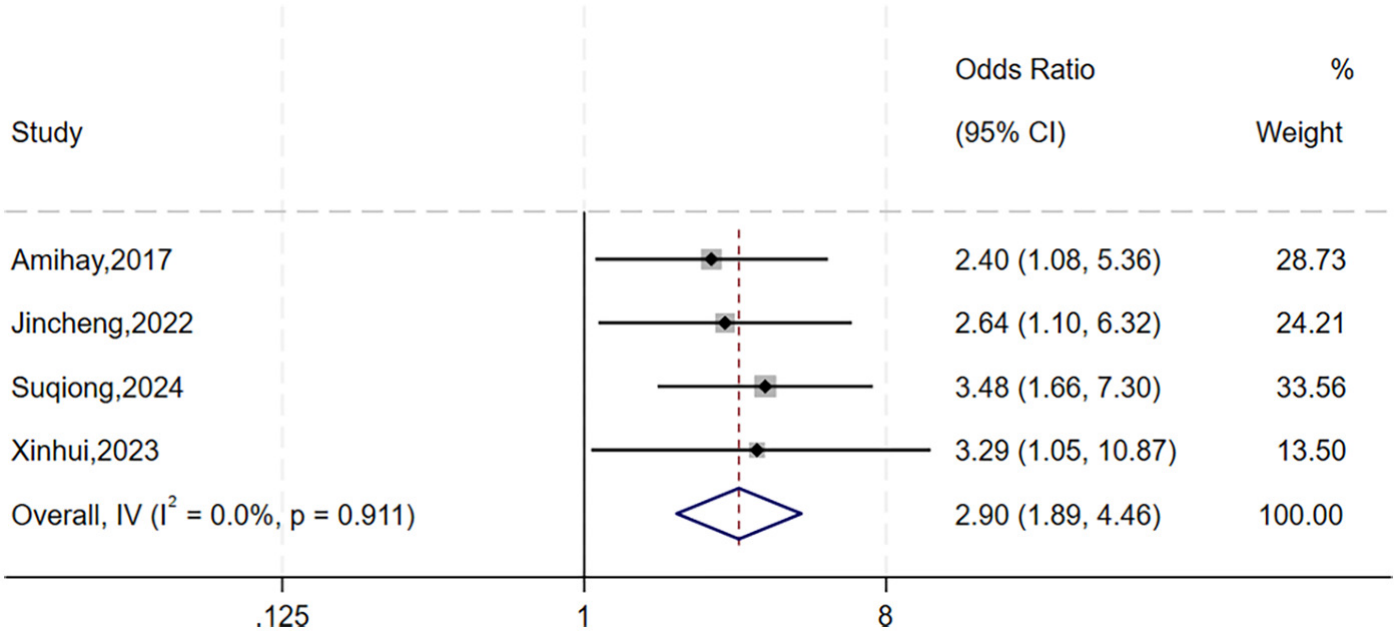
Leave-one-out sensitivity forest plot of gender.

The heterogeneity test for gender showed significant variation across studies (*I*^2^ = 81%, *P* = 0.001). As subgroup analysis was not feasible, we performed sensitivity analysis by sequentially excluding individual studies (Figure 2). The study by Xiaobo et al. (12) was identified as the primary source of heterogeneity. After excluding this study, heterogeneity was significantly reduced (*I*^2^ = 0%, *P* = 0.911), and a fixed-effects model was applied. The analysis revealed that gender was significantly with higher risks of urosepsis following URSL (OR = 2.90, 95% CI: 1.89, 4.46; *P* < 0.001).

**Figure 3:**
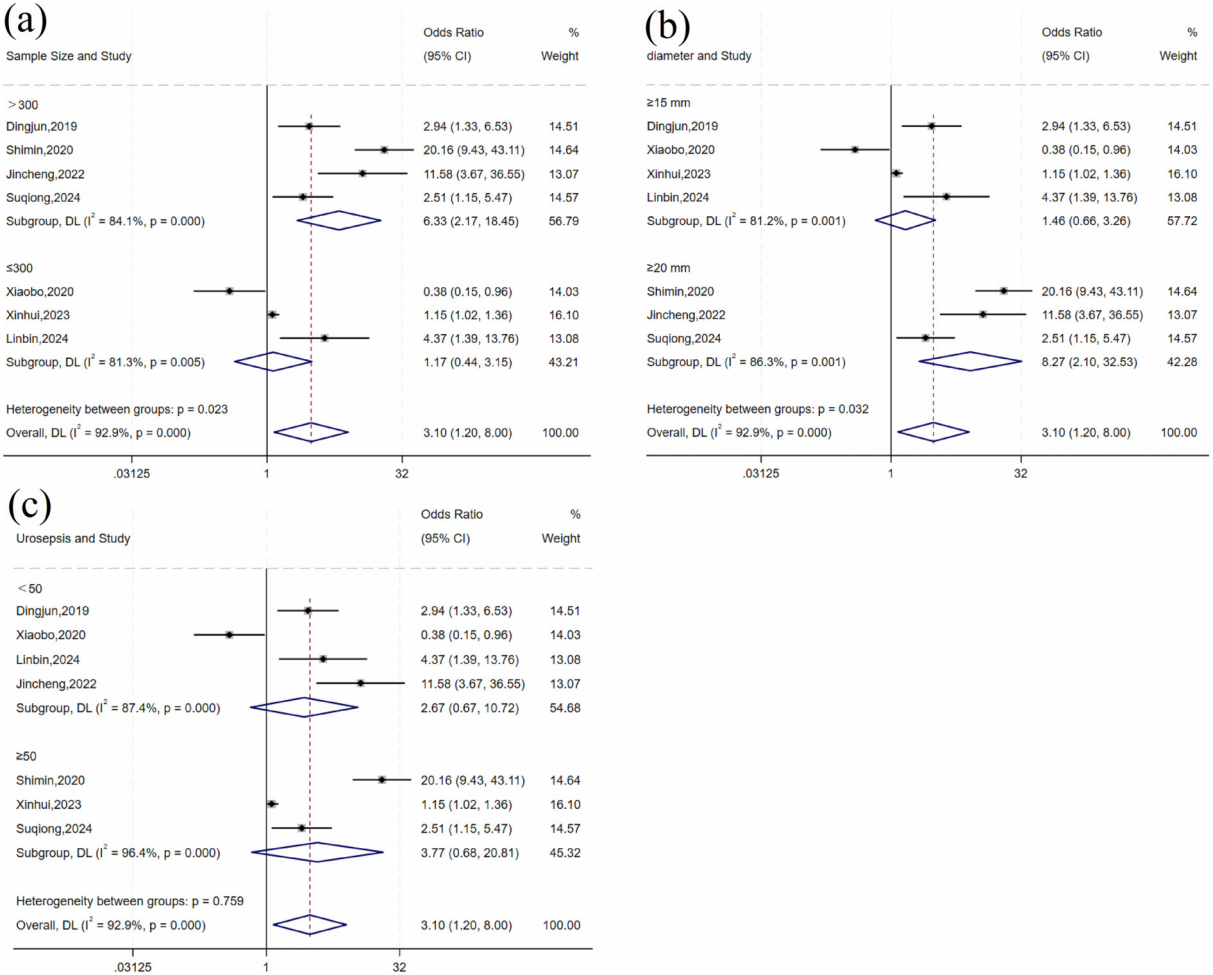
Forest plot of subgroup analysis of stone size: **(a)** sample size; **(b)** diameter; **(c)** number of urosepsis cases.

The heterogeneity test for stone size showed significant variation across studies (*I*^2^ = 92.9%, *P* = 0.001). Subgroup analyses by sample size, stone diameter, and number of urosepsis cases (Table 3 and Figure 3) revealed significant between-group differences by sample size (*P* = 0.001) and stone diameter (*P* = 0.032), suggesting these factors may influence urosepsis risk after URSL. However, substantial heterogeneity persisted within these subgroups, indicating that other sources of variation remain unaccounted for.

**Table 3:** Subgroup analysis for stone size factors.

Subgroup	Number of studies	Heterogeneity test		Effect model	Pooled effect		*P*-value for between-group differences
		*I*^2^ (%)	*P* value		OR (95% CI)	*P* value	
Sample size							
≤300	3	81.3	0.005	Random	1.17 (0.44, 3.15)	0.754	0.023
>300	4	84.1	<0.001	Random	6.33 (2.17, 18.45)	0.001	
Stone diameter (mm)							
≥15	4	81.2	0.09	Random	1.46 (0.66, 3.26)	0.349	0.032
≥20	3	86.3	<0.001	Random	8.27 (2.10, 32.53)	0.002	
Number of US							
<50	4	87.4	<0.001	Random	2.67 (0.67, 10.72)	0.166	0.759
≥50	3	96.4	<0.001	Random	3.77 (0.68, 20.81)	0.128	

**Figure 4:**
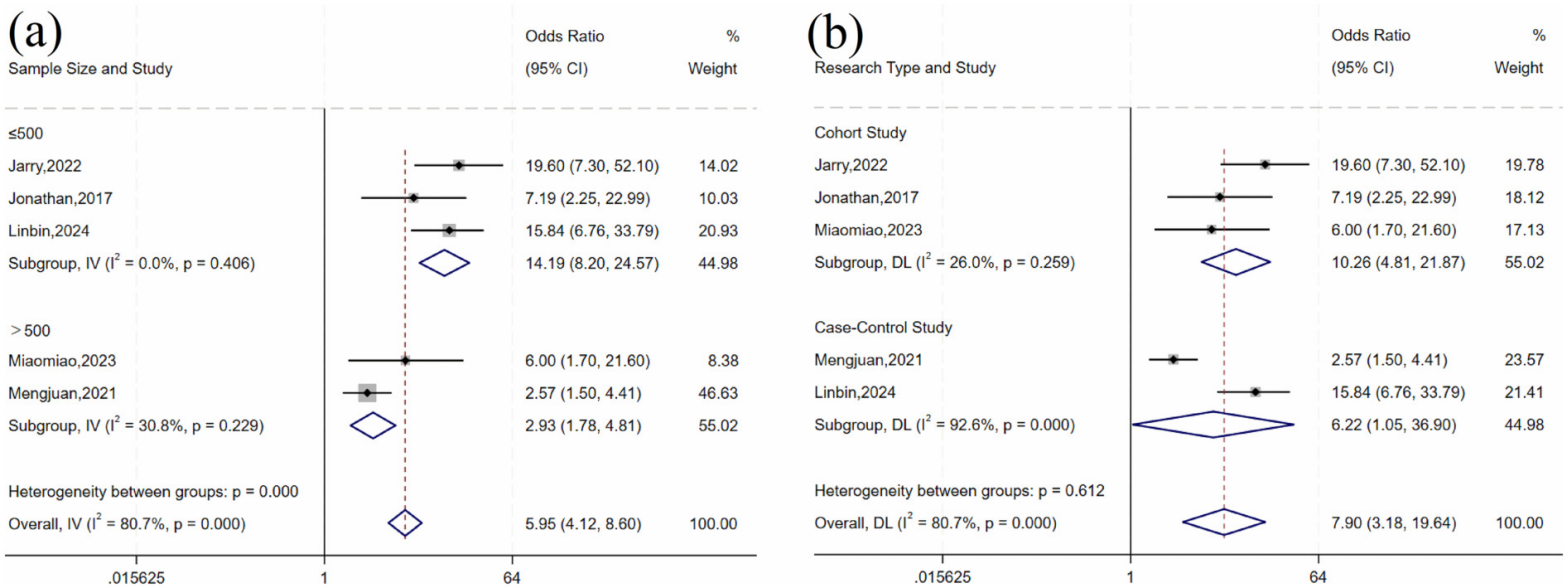
Forest plot of subgroup analysis of UTI history: **(a)** sample size; **(b)** research type.

For history of UTI, there was heterogeneity across studies (*I*^2^ = 80.7%, *P* = 0.001). Subgroup analysis by sample size and study design (Table 4 and Figure 4) showed statistically significant differences by sample size subgroups (*P* = 0.032). Heterogeneity was significantly reduced in both subgroups (≤500 group: *I*^2^ = 0%, *P* = 0.406; >500 group: *I*^2^ = 30.8%, *P* = 0.229), suggesting that sample size may influence the association between UTI history and risk of urosepsis after URSL. Fixed-effects model analysis showed a statistically significant pooled effect (OR = 5.96, 95% CI: 4.12, 8.60; *P* < 0.001).

**Table 4:** Subgroup analysis for UTI factors.

Subgroup	Number of studies	Heterogeneity test		Effect model	Pooled effect		*P*-value for between-group differences
		*I*^2^ (%)	*P* value		OR (95% CI)	*P* value	
Sample size							
≤500	3	0	0.406	Fixed	14.19 (8.20, 24.57)	<0.001	0.001
>500	2	30.8	0.229	Fixed	2.93 (1.78, 4.81)	<0.001	
Study type							
Cohort study	3	26	0.259	Random	10.26 (4.81, 21.87)	<0.001	0.612
Case-control study	2	92.6	<0.001	Random	6.22 (1.05, 36.90)	0.044	

**Figure 5:**
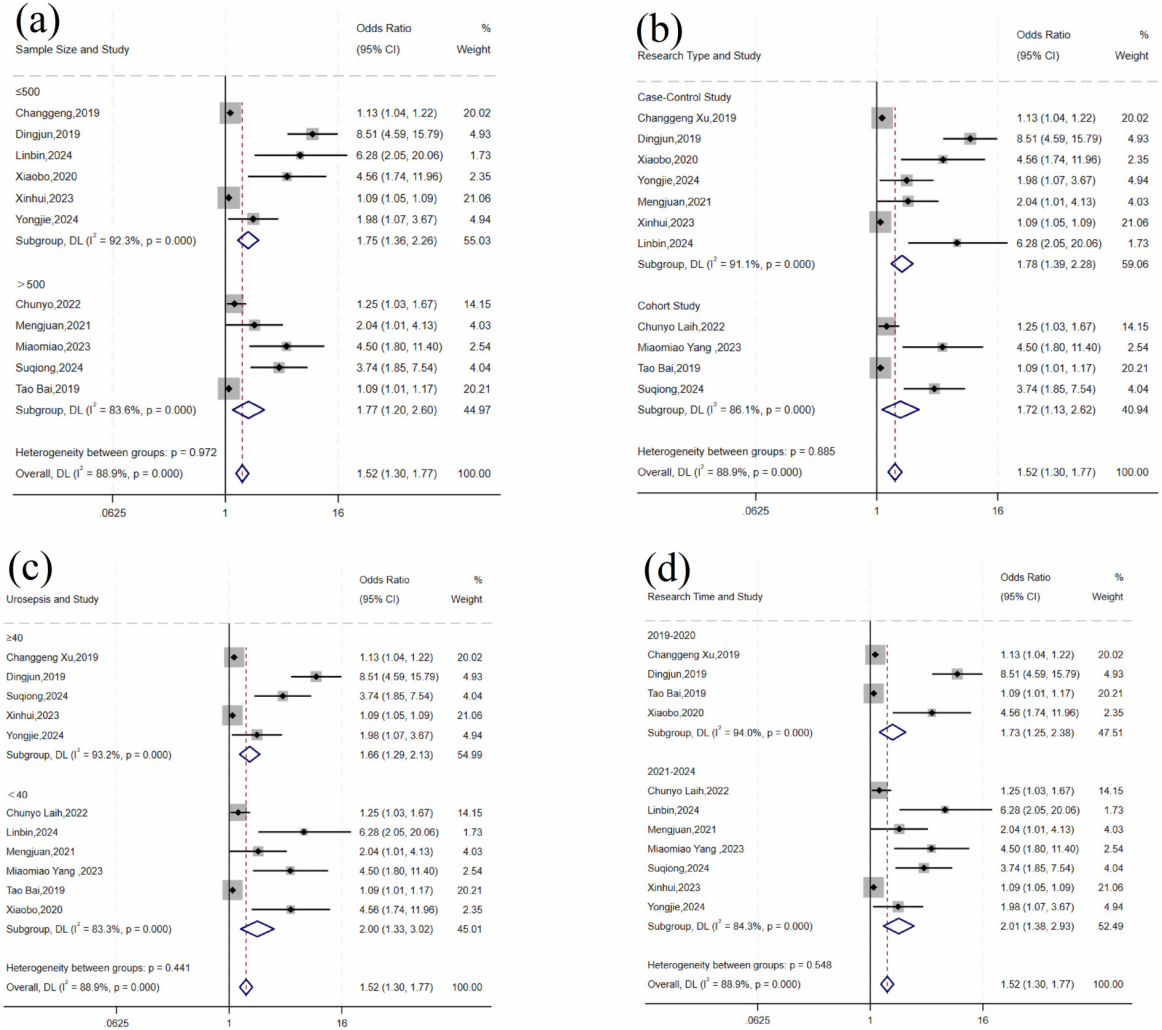
Forest plot of subgroup analysis of operative time: **(a)** sample size; **(b)** research type; **(c)** number of urosepsis cases; **(d)** research time.

For operative time, there was significant variation across studies (*I*^2^ = 88.9%, *P* < 0.001). Subgroup analyses by sample size, study design, number of urosepsis cases, and publication year did not significantly reduce heterogeneity (Table 5 and Figure 5). Sensitivity analysis by sequentially excluding individual studies identified five studies (11, 12, 16, 17, 28) as the primary sources of heterogeneity. After excluding these studies, heterogeneity was significantly reduced (*I*^2^ = 43.8%, *P* = 0.113). Fixed-effects meta-analysis demonstrated a statistically significant pooled effect (OR = 1.09, 95% CI: 1.07, 1.11; *P* < 0.001).

**Table 5:** Subgroup analysis for operative time factors.

Subgroup	Number of studies	Heterogeneity test		Effect model	Pooled effect		*P-*value for between-group differences
		*I*^2^ (%)	*P* value		OR (95% CI)	*P* value	
Sample size							
≤500	6	92.3	<0.001	Random	1.75 (1.36, 2.26)	<0.001	0.972
>500	5	83.6	<0.001	Random	1.77 (1.20, 2.60)	0.004	
Study type							
Case-control study	7	91.1	<0.001	Random	1.78 (1.40, 2.29)	<0.001	0.885
Cohort study	4	86.1	<0.001	Random	1.72 (1.13, 2.62)	0.012	
Number of US							
<40	6	83.3	<0.001	Random	2.00 (1.33, 3.02)	0.001	0.441
≥40	5	93.2	<0.001	Random	1.66 (1.29, 2.13)	<0.001	
Year of study							
2019–2020	4	94	<0.001	Random	1.73 (1.25, 2.38)	0.001	0.548
2021–2024	7	84.3	<0.001	Random	2.01 (1.38, 2.93)	<0.001	

### 3.6 Publication bias

**Figure 6:**
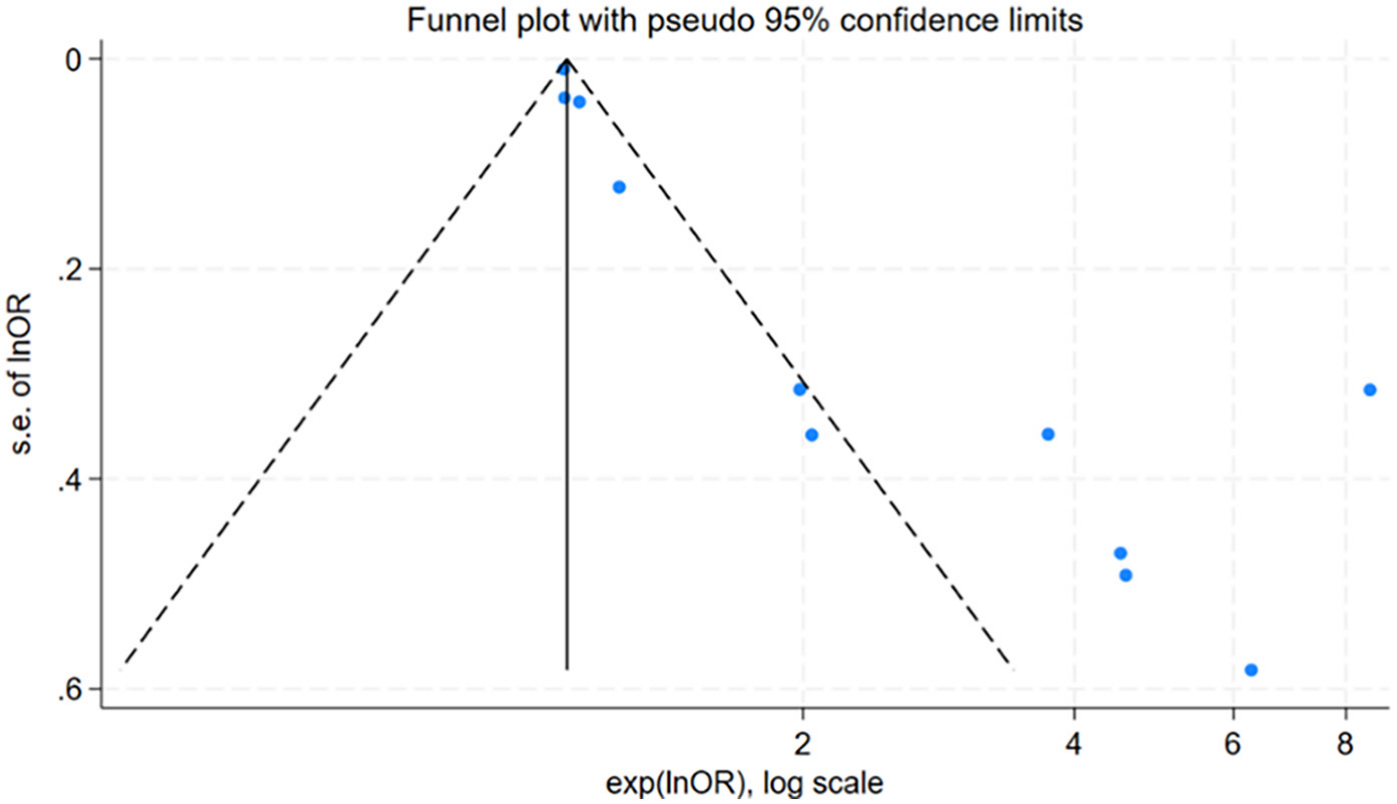
Funnel plot of operative time.

**Figure 7:**
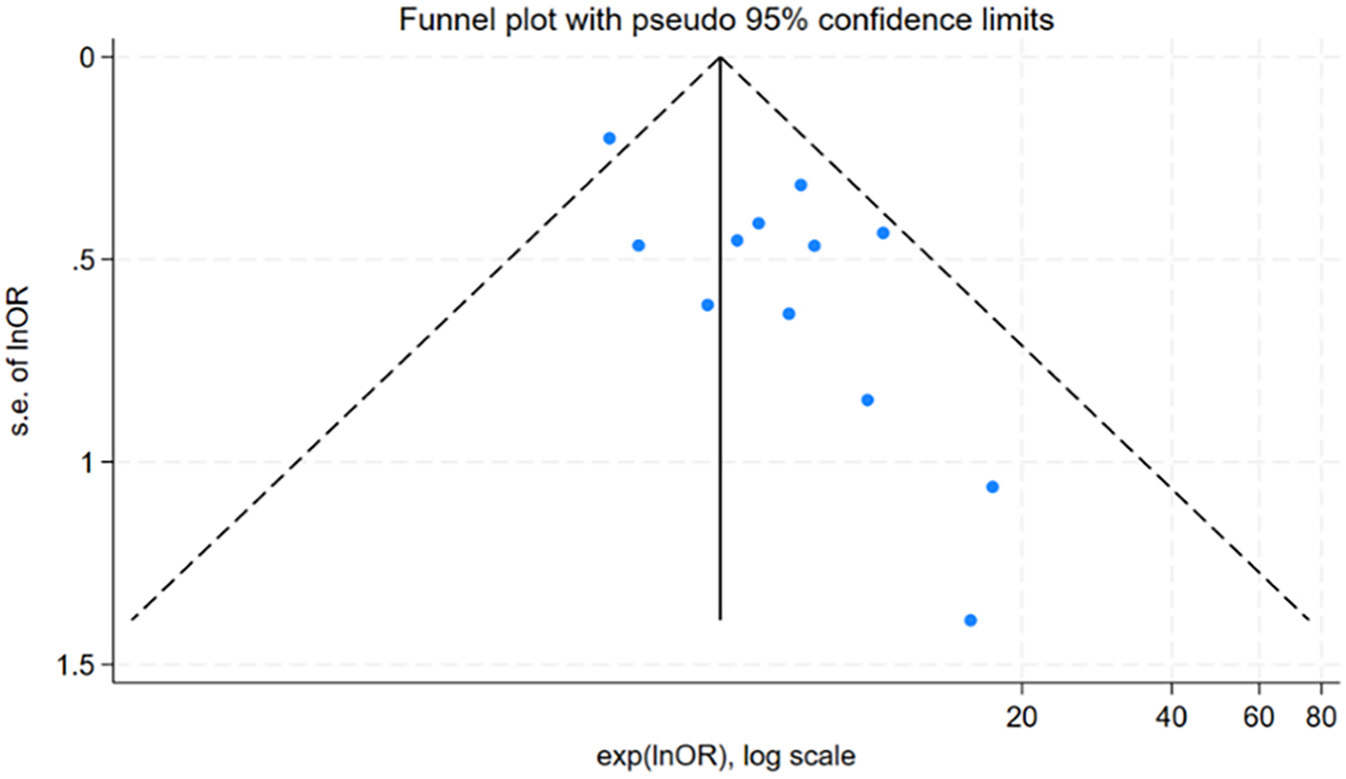
Funnel plot of positive urine culture.

For factors reported in more than 10 studies, publication bias was assessed using funnel plots (Figure 6 and Figure 7) and Egger's test (Table 6). The findings revealed potential publication bias for operation time (*P* = 0.001) and positive urine culture (*P* = 0.013). The trim-and-fill method was applied using Stata 15.0 to correct for potential bias.

**Table 6:** Publication bias assessment for risk factors of urosepsis after ureteroscopic lithotripsy.

Factors	Egger's test	Trim-and-fill number	Combined effect size before trim-and-fill		Combined effect size after trim-and-fill	
	*P* value		OR (95% CI)	*P* value	OR (95% CI)	*P* value
Operation time	0.001	6	0.48 (0.26, 0.57)	<0.001	1.15 (0.96, 1.37)	0.133
Positive urine culture	0.013	6	1.60 (1.37, 1.83)	<0.001	3.77 (3.07, 4.63)	<0.001

For surgical time, the adjusted effect size (OR = 1.15; 95% CI: 0.96, 1.37; *P* = 0.133), differing substantially from the original estimate (OR = 0.48, 95% CI: 0.26, 0.57); *P* < 0.001), suggesting significant publication bias, indicating that the original results may have been affected by unpublished studies. In contrast, the adjusted effect size for positive urine culture (OR = 3.77, 95% CI: 3.07, 4.63; *P* < 0.001) was relatively consistent with the original findings (OR = 1.60 95% CI: 1.37, 1.83; *P* < 0.001), suggesting the initial results were robust despite some publication bias.

## 4 Discussion

### 4.1 General demographic and disease-related factors

The findings of this meta-analysis demonstrated that diabetes mellitus was significantly associated with urosepsis post-URSL, consistent with the findings of Sun et al. (29), which revealed an association between diabetes mellitus and the risk of infection. Hyperglycemia in diabetes mellitus not only provides an energy source for bacterial growth but also significantly dysregulates both innate and adaptive immune responses, increasing susceptibility to infections. Innate immune dysfunction includes impaired neutrophil chemotaxis, phagocytosis, and oxidative burst, reduced complement activation, and NK cell cytotoxicity. Adaptive immunity is suppressed by defective antigen-presenting cell (APC) maturation, diminished cytokine production, CD4+ T-cell dysfunction, and disrupted B-cell antibody production (30, 31). These immune and metabolic alterations account for the increased infection risk in diabetic patients following URSL.

Although gender was not statistically significant in this meta-analysis, Dan et al. (32) have reported that gender significantly impacts the risk of infection following URSL. Potential reasons for women's increased susceptibility to urosepsis after URSL may include genetic factors and effects of sex hormones (29). Genetic differences can affect immune system function and infection resistance by altering how immune-related genes work. Female sex hormones like estrogen and progesterone may also influence inflammation and immune responses by changing how immune cells function and produce cytokines, which could affect the body's ability to fight infections. Assessment of preoperative infection risk is essential in female patients and patients with diabetes undergoing URSL. Appropriate preventive measures, such as glycemic control, should be implemented to reduce the risk of postoperative urosepsis.

### 4.2 Stone-related factors

The meta-analysis found that stone size and stone number were significantly associated with the risk of urosepsis following URSL. Although flexible ureteroscopy has been demonstrated to be effective for renal calculi >2 cm (33), Jeong et al. (34) found that stone diameter ≥1.5 cm is associated with increased infection risk. The duration of lithotripsy is significantly prolonged in cases involving larger or multiple urinary stones. Continuous laser energy and mechanical forces break down the stone structure, releasing trapped endotoxins and bacteria (35). During the procedure, the necessary high-pressure irrigation increases the permeability of small blood vessels and lymph channels of the kidney, creating pathways for bacteria to enter the bloodstream. Additionally, larger stones can cause urinary tract obstruction (36), impairing urine drainage and promoting bacterial growth. These physiological changes allow bacteria and their toxic products to spread throughout the body, significantly increasing the risk of serious infection. For patients with large or multiple stones, preoperative infection risk assessment is crucial, and lithotripsy time should be minimized to reduce postoperative infections.

### 4.3 Infection-related factors

This study found that a history of UTI, positive urine culture, positive urinary nitrite, and elevated CRP levels were significantly associated with urosepsis following URSL. Persistent fever typically indicates the presence of pro-inflammatory factors and uncontrolled infection. Inflammation in the urinary tract induces mucosal edema, vasodilation, and compromised vascular barrier function, thereby disrupting the natural mucosal defense and increasing the risk of bacterial and toxin translocation into the circulation. Francesco et al. (37) demonstrated that prolonged URSL operation time is significantly associated with an elevated risk of postoperative UTI. Urine culture positivity is the gold standard for diagnosing UTI, providing direct evidence of viable bacteria in the urine. A meta-analysis by Naeem et al. (38) demonstrated that a positive urine culture is a risk factor for sepsis, with microbiological analysis identifying *Escherichia coli* as the predominant uropathogen (39). A positive nitrite result indicates the reduction of nitrate to nitrite by Gram-negative bacteria in urine. This biochemical conversion typically requires a high bacterial load, and such significant bacteriuria frequently triggers pronounced inflammatory responses. CRP serves as a sensitive systemic inflammation marker that can increase up to 1,000-fold at infection or inflammation sites (40). The non-significant association for procalcitonin in this study may be attributed to the limited number of included studies. Villanueva et al. (41) reported that leukocyte counts typically increase during bacterial UTIs. The inconsistent findings in this study may be because severe infections can show an initial decrease followed by an increase due to endotoxin-mediated leukocyte depletion (42). Notably, the associations for both procalcitonin (OR = 9.34, 95% CI: 0.1–878.49; *P* = 0.335) and positive urine nitrite (OR = 7.68, 95% CI: 1.03–52.27; *P* < 0.001) had markedly wide confidence intervals, reflecting population heterogeneity and methodological differences. Thus, we strongly recommend cautious interpretation of these biomarkers in clinical practice. Therefore, UTIs should be treated before lithotripsy. Antibiotic therapy should be guided by preoperative urine culture (43), and prophylactic antibiotics may also help reduce infection risk in patients with negative urine cultures.

### 4.4 Surgery-related factors

We found that procedure duration and stent placement were significantly associated with urosepsis post-URSL. Ozgor et al. (44) reported that procedures lasting more than one hour doubled the risk of infectious complications, as prolonged exposure increases the likelihood of pathogenic bacteria and endotoxins entering the bloodstream. Preoperative double J stent placement improves stone clearance in patients undergoing URSL. However, a meta-analysis by Corrales et al. (45) demonstrated that stent retention exceeding 30 days increases sepsis rates. Prolonged stenting may induce chronic inflammation, causing local tissue damage and persistent release of inflammatory factors, thereby elevating sepsis risk. Therefore, although preoperative stent placement is recommended, its duration should be carefully monitored. Additionally, controlled procedure times are essential during surgery.

### 4.5 Study limitations

This study has several limitations: (1) The inclusion of only Chinese and English literature may introduce language bias; (2) Most included studies were of moderate quality, highlighting the need for additional high-quality research; (3) Significant heterogeneity was observed in some influencing factors—although subgroup analyses identified partial sources, other heterogeneity factors remain to be elucidated; (4) Some risk factors were reported in single studies, limiting the reliability of these findings due to insufficient data.

## 5 Conclusion

Urosepsis, although relatively rare, represents the most severe complication following URSL and is associated with significant mortality rates. This study employed meta-analysis to identify risk factors for urosepsis after URSL. The analysis revealed that stone size, stone number, history of UTI, positive urine culture, positive urinary nitrites, elevated CRP levels, prolonged operative time, stent placement, and diabetes mellitus were significantly associated with postoperative US. These findings provide valuable insights for developing early identification and treatment strategies. Future research should focus on creating predictive models and web-based calculators to improve clinical risk assessment, which may enhance patient outcomes and optimize healthcare resource utilization.

## References

[1] Cook J Lamb BW Lettin JE Graham SJ. The epidemiology of urolithiasis in an ethnically diverse population living in the same area. Urol J. (2016) 13(4):2754–8. 10.22037/uj.v13i4.3336 27576881

[2] De S Autorino R Kim FJ Zargar H Laydner H Balsamo R Percutaneous nephrolithotomy versus retrograde intrarenal surgery: a systematic review and meta-analysis. Eur Urol. (2015) 67(1):125–37. 10.1016/j.eururo.2014.07.003 25064687

[3] Doizi S Traxer O. Flexible ureteroscopy: technique, tips and tricks. Urolithiasis. (2018) 46(1):47–58. 10.1007/s00240-017-1030-x 29222575

[4] Tang QL Liang P Ding YF Zhou XZ Tao RZ. Comparative efficacy between retrograde intrarenal surgery with vacuum-assisted ureteral access sheath and minimally invasive percutaneous nephrolithotomy for 1–2 cm infectious upper ureteral stones: a prospective, randomized controlled study. Front Surg. (2023) 10:1200717. 10.3389/fsurg.2023.1200717 37483661 PMC10360123

[5] Inoue T Okada S Hamamoto S Fujisawa M. Retrograde intrarenal surgery: past, present, and future. Investig Clin Urol. (2021) 62(2):121–35. 10.4111/icu.20200526 33660439 PMC7940851

[6] Page MJ McKenzie JE Bossuyt PM Boutron I Hoffmann TC Mulrow CD The PRISMA 2020 statement: an updated guideline for reporting systematic reviews. BMJ. (2021) 372:n71. 10.1136/bmj.n71 33782057 PMC8005924

[7] Kaczmarek K Jankowska M Kalembkiewicz J Kienitz J Chukwu O Lemiński A Assessment of the incidence and risk factors of postoperative urosepsis in patients undergoing ureteroscopic lithotripsy. Cent European J Urol. (2024) 77(1):122. 10.5173/ceju.2023.167 38645806 PMC11032028

[8] Guliciuc M Maier AC Maier IM Kraft A Cucuruzac RR Marinescu M The urosepsis-a literature review. Medicina (Kaunas). (2021) 57(9):872. 10.3390/medicina57090872 34577795 PMC8468212

[9] Stang A. Critical evaluation of the Newcastle-Ottawa scale for the assessment of the quality of nonrandomized studies in meta-analyses. Eur J Epidemiol. (2010) 25(9):603–5. 10.1007/s10654-010-9491-z 20652370

[10] Chen W He Y Lu K Liu C Jiang T Zhang H Construction of a back propagation neural network model for predicting urosepsis after flexible ureteroscopic lithotripsy. J Zhejiang Univ Med Sci. (2025) 54(01):1–9. 10.3724/zdxbyxb-2024-0128 PMC11956881 39647846

[11] Ding J Zhou C Li G Chen G Zhu X Liu J Influencing factors for postoperative urine-induced sepsis in flexible ureteroscope lithotripsy patients. Chinese J Nosocomiol. (2019) 29(14):2134–7+42. 10.11816/cn.ni.2019-181950

[12] Hu X Tang Q. Risk factors of urosepsis after flexible ureteroscopic holmium Laser lithotripsy. Chinese Gen Pract. (2020) 23(S1):86–8. https://kns.cnki.net/kcms2/article/abstract?v=6WbqZcVy3rg_sY-78zNPpR7Xa1DaxaEb-dpnr60ohIb4ZXEdxO59bxYCbLuxBQ5bkS6dcw0i3OFfRb6-eNThqHRbGvcKrXqFwPoBuruyZ7PCv0Hok91AlBTVb6hKJQ4amEFcMvd56bjzrNORcuXYmXAzeISXYE5jGGSPp4kkvuA=&uniplatform=NZKPT

[13] Ma S Huang Y Yang M. Analysis of influencing factor of urinary sepsis in patients undergoing retrograde intrarenal surgery under flexible ureteroscope. China Med Herald. (2022) 19(27):76–9. 10.20047/j.issn1673-7210.2022.27.17

[14] Yin J Gu Y Tang X Guo Z Liu H. Risk factors for urosepsis after retrograde intrarenal surgery and construction of a nomogram prediction model. J Minimally Invasive Urol. (2022) 11(04):239–45. 10.19558/j.cnki.10-1020/r.2022.04.006

[15] Xinhui W Liangzhi Y Yanhua Y. Study on the influencing factors of urinary sepsis after ureteral flexible lithotripsy. Shenzhen J Integr Traditional Chinese Western Med. (2023) 33(12):15–8. 10.16458/j.cnki.1007-0893.2023.12.005

[16] Wu L Chen W Yang J Chen D. Analysis of risk factors for urosepsis after flexible ureteroscopic lithotripsy. Zhejiang J Clin Med. (2024) 26(04):530–1. 10.3969/j.issn.1008-7664.2024.4.zjlcyx202404018

[17] Zhang S He D Zhao X. Construction and validation of a risk prediction model for postoperative urogenic sepsis in patients with ureteral calculi. Evid Based Nurs. (2024) 10(16):2913–8. 10.12102/j.issn.2095-8668.2024.16.013

[18] Meng J Cai H Zhang X Yang H Huang Y. Influencing factors of urinary sepsis in patients with ureteral calculi and diagnostic value of peripheral blood Nlr, Plr And Pct. Chinese J Nosocomiol. (2021) 31(20):3082–5. 10.11816/cn.ni.2021-203764

[19] Ma Y Deng S Wei X Wu Z Wei W. Risk factors for postoperative urogenic sepsis in patients undergoing ureteroscopy with holmium Laser lithotripsy and changes of Tlr4/nf-Κb signaling pathways. Chinese J Nosocomiol. (2024) 34(02):235–8. 10.11816/cn.ni.2024-230795

[20] Blackmur JP Maitra NU Marri RR Housami F Malki M McIlhenny C. Analysis of factors’ association with risk of postoperative urosepsis in patients undergoing ureteroscopy for treatment of stone disease. J Endourol. (2016) 30(9):963–9. 10.1089/end.2016.0300 27317017

[21] Wang C Xu R Zhang Y Wu Y Zhang T Dong X Nomograms for predicting the risk of sirs and urosepsis after uroscopic minimally invasive lithotripsy. Biomed Res Int. (2022) 2022:6808239. 10.1155/2022/6808239 35309171 PMC8933078

[22] Jarry É Garot M Marlière F Fantoni J-C Villers A Lebuffe G Predictive factors of postoperative septic complications after flexible ureteroscopy for urinary stones. Prog Urol. (2022) 32(2):85–91. 10.1016/j.purol.2021.07.010 34509371

[23] Xu CG Guo YL. Diagnostic and prognostic values of bmper in patients with urosepsis following ureteroscopic lithotripsy. BioMed Res Int. (2019) 2019(1):8078139. 10.1155/2019/8078139 30800678 PMC6360565

[24] Wang L Yu X Qiu Z Liu P Tian W He W Influence of preoperative urine culture and bacterial Species on urogenital sepsis after ureteral flexible lithotripsy in patients with upper urinary tract stones. Front Med (Lausanne). (2024) 11:1393734. 10.3389/fmed.2024.1393734 38765255 PMC11099900

[25] Devos B Vanderbruggen W Claessens M Duchateau A Hente R Keller EX Risk factors of early infectious complications after ureterorenoscopy for stone disease: a prospective study. World J Urol. (2024) 42(1):277. 10.1007/s00345-024-04983-6 38691160

[26] Nevo A Mano R Baniel J Lifshitz DA. Ureteric stent dwelling time: a risk factor for post-ureteroscopy sepsis. BJU Int. (2017) 120(1):117–22. 10.1111/bju.13796 28145037

[27] Laih C-Y Hsiao P-J Hsieh P-F Wang Y-D Lai C-M Yang C-T Qsofa and Sofa scores are valuable tools for predicting postoperative sepsis resulting from ureteroscopic lithotripsy (ursl). Medicine (Baltimore). (2022) 101(50):e31765. 10.1097/MD.0000000000031765 36550908 PMC9771339

[28] Yang M Li Y Huang F. A nomogram for predicting postoperative urosepsis following retrograde intrarenal surgery in upper urinary calculi patients with negative preoperative urine culture. Sci Rep. (2023) 13(1):2123. 10.1038/s41598-023-29352-y 36747018 PMC9902470

[29] Sun J Xu J OuYang J. Risk factors of infectious complications following ureteroscopy: a systematic review and meta-analysis. Urol Int. (2020) 104(1-2):113–24. 10.1159/000504326 31846966

[30] Berbudi A Rahmadika N Tjahjadi AI Ruslami R. Type 2 diabetes and its impact on the immune system. Curr Diabetes Rev. (2020) 16(5):442–9. 10.2174/1573399815666191024085838 31657690 PMC7475801

[31] Velloso LA Eizirik DL Cnop M. Type 2 diabetes mellitus–an autoimmune disease? Nat Rev Endocrinol. (2013) 9(12):750–5. 10.1038/nrendo.2013.131 23835371

[32] Tan D Wu F Huo W. Clinical characteristics and risk factors of systemic inflammatory response syndrome after flexible ureteroscopic lithotripsy. Arch Esp Urol. (2022) 75(7):618–23. 10.56434/j.arch.esp.urol.20227507.89 36214143

[33] Cosmin C Georgescu DA Geavlete P Popescu RI Geavlete B. Comparison between retrograde flexible ureteroscopy and percutaneous nephrolithotomy for the treatment of renal stones of 2–4 cm. Medicina (Kaunas). (2023) 59(1):124. 10.3390/medicina59010124 36676748 PMC9864526

[34] Yoo JW Lee KS Chung BH Kwon SY Seo YJ Lee KS Optimal duration of preoperative antibiotic treatment prior to ureteroscopic lithotripsy to prevent postoperative systemic inflammatory response syndrome in patients presenting with urolithiasis-induced obstructive acute pyelonephritis. Investig Clin Urol. (2021) 62(6):681–9. 10.4111/icu.20210160 34387040 PMC8566789

[35] Ye S Wang W Yu Z Luo J. Risk factors for systemic inflammatory response syndrome after endoscopic lithotripsy for upper urinary calculi. BMC Urol. (2023) 23(1):59. 10.1186/s12894-023-01230-9 37041554 PMC10091591

[36] Heijkoop B Galiabovitch E York N Webb D. Consensus of multiple national guidelines: agreed strategies for initial stone management during COVID-19. World J Urol. (2021) 39(9):3161–74. 10.1007/s00345-020-03491-7 33226444 PMC7681178

[37] Prata F Cacciatore L Salerno A Tedesco F Ragusa A Basile S Urinary tract infection predictors in patients undergoing retrograde intrarenal surgery for renal stones: does the instrument make the difference? J Clin Med. (2024) 13(10):2758. 10.3390/jcm13102758 38792300 PMC11122071

[38] Bhojani N Miller LE Bhattacharyya S Cutone B Chew BH. Risk factors for urosepsis after ureteroscopy for stone disease: a systematic review with meta-analysis. J Endourol. (2021) 35(7):991–1000. 10.1089/end.2020.1133 33544019

[39] Scotland KB Lange D. Prevention and management of urosepsis triggered by ureteroscopy. Res Rep Urol. (2018) 10:43–9. 10.2147/rru.S128071 30013956 PMC6038880

[40] Sproston NR Ashworth JJ. Role of C-reactive protein at sites of inflammation and infection. Front Immunol. (2018) 9:754. 10.3389/fimmu.2018.00754 29706967 PMC5908901

[41] Villanueva-Congote J Hinojosa-Gonzalez D Segall M Eisner BH. The relationship between neutrophil/lymphocyte ratio, platelet/neutrophil ratio, and risk of urosepsis in patients who present with ureteral stones and suspected urinary tract infection. World J Urol. (2024) 42(1):596. 10.1007/s00345-024-05229-1 39466513

[42] Huang J Xie L Yang Y Xie H Liu C. Hypoalbuminemia within one hour after surgery as a predictor of post-operative urosepsis in patients undergoing flexible ureteroscopy lithotripsy: a retrospective study. Surg Infect (Larchmt). (2023) 24(1):75–81. 10.1089/sur.2022.290 36579922

[43] Khusid JA Hordines JC Sadiq AS Atallah WM Gupta M. Prevention and management of infectious complications of retrograde intrarenal surgery. Front Surg. (2021) 8:718583. 10.3389/fsurg.2021.718583 34434958 PMC8381273

[44] Ozgor F Sahan M Cubuk A Ortac M Ayranci A Sarilar O. Factors affecting infectious complications following flexible ureterorenoscopy. Urolithiasis. (2019) 47(5):481–6. 10.1007/s00240-018-1098-y 30448869

[45] Corrales M Sierra A Doizi S Traxer O. Risk of sepsis in retrograde intrarenal surgery: a systematic review of the literature. Eur Urol Open Sci. (2022) 44:84–91. 10.1016/j.euros.2022.08.008 36071820 PMC9442387

[46] Sun J-X Xu J-Z Liu C-Q Xun Y Lu J-l Xu M-Y A novel nomogram for predicting post-operative sepsis for patients with solitary, unilateral and proximal ureteral stones after treatment using percutaneous nephrolithotomy or flexible ureteroscopy. Front Surg. (2022) 9:814293. 10.3389/fsurg.2022.814293 35495750 PMC9051077

[47] Bloom J Fox C Fullerton S Matthews G Phillips J. Sepsis after elective ureteroscopy. Can J Urol. (2017) 24(5):9017–23. https://pubmed.ncbi.nlm.nih.gov/28971790/28971790

[48] Lu J Xun Y Yu X Liu Z Cui L Zhang J Albumin-globulin ratio: a novel predictor of sepsis after flexible ureteroscopy in patients with solitary proximal ureteral stones. Transl Androl Urol. (2020) 9(5):1980–9. 10.21037/tau-20-823 33209662 PMC7658149

[49] Pattou M Yonneau L de Gouvello A Almeras C Saussine C Hoznek A Urosepsis after ureterorenoscopy, intraoperative recognition of type-iv stones could change clinical practice. World J Urol. (2024) 42(1):534. 10.1007/s00345-024-05251-3 39306607

[50] Bai T Yu X Qin C Xu T Shen H Wang L Identification of factors associated with postoperative urosepsis after ureteroscopy with holmium: yttrium-aluminum-garnet Laser lithotripsy. Urol Int. (2019) 103(3):311–7. 10.1159/000502159 31461729

