# Supplementary material for: Risk factors for urosepsis following ureteroscopic lithotripsy: a systematic review and meta-analysis
Source: Front Surg. 2025 Jun 19;12:1603311. doi: 10.3389/fsurg.2025.1603311 (PMC12222153; doi:10.3389/fsurg.2025.1603311)
Supplement: Supplementary file 1 [file Datasheet1.docx]

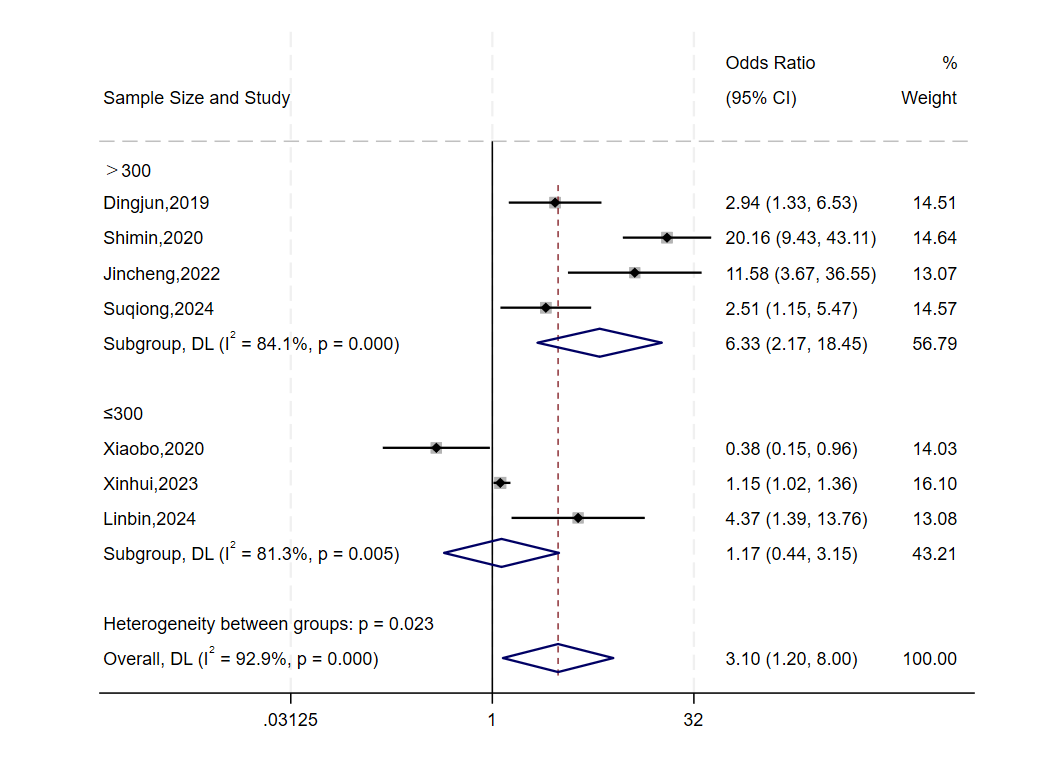


**Figure 1：Stone size - Subgroup analysis - Sample Size**


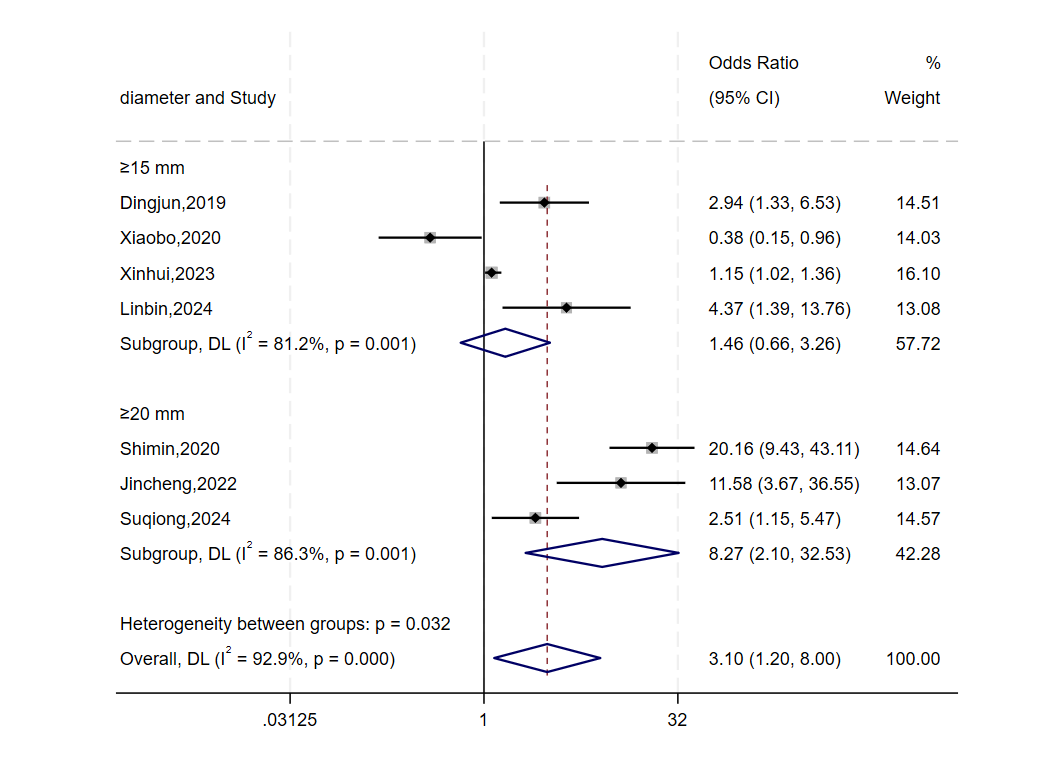


**Figure 2：Stone size - Subgroup analysis – diameter**

**
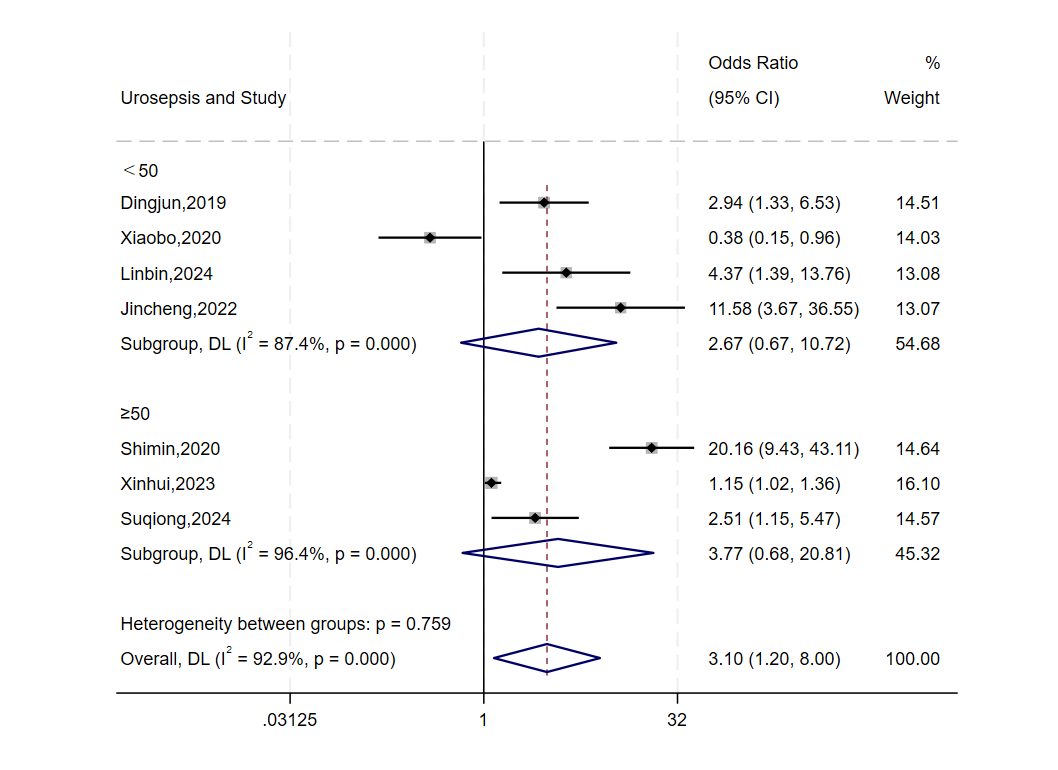
**

**Figure 3：Stone size - Subgroup analysis – Urosepsis**

**
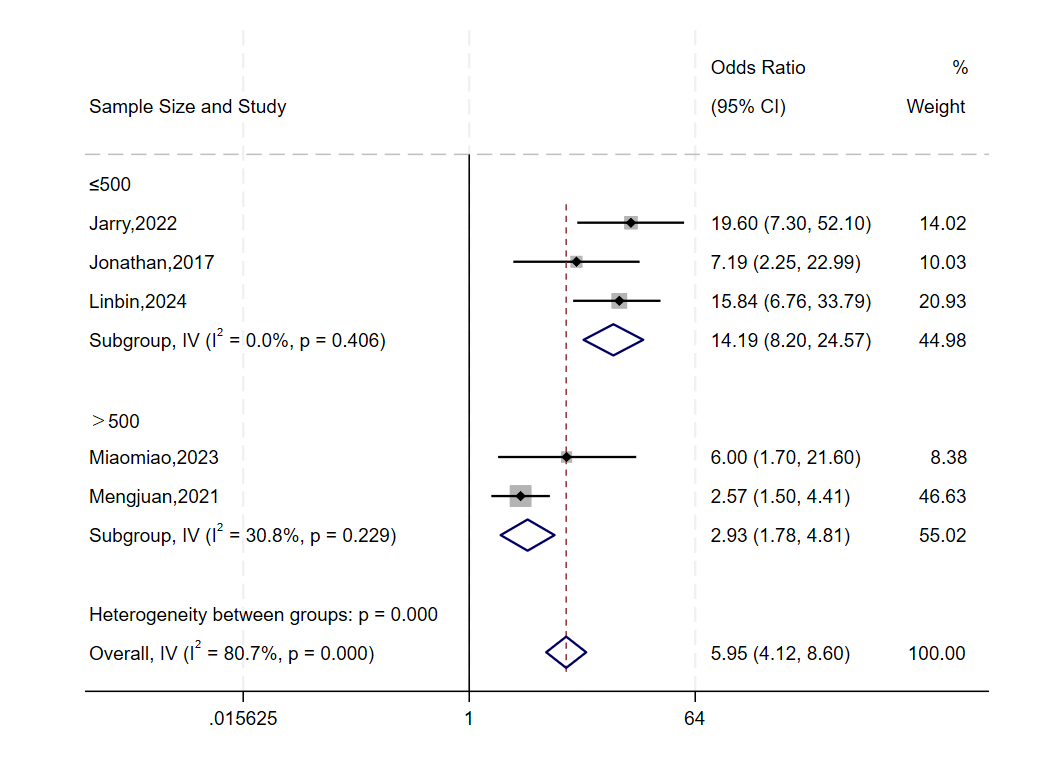
**

**Figure 4：History of urinary tract infection- Subgroup analysis -Sample Size**

**
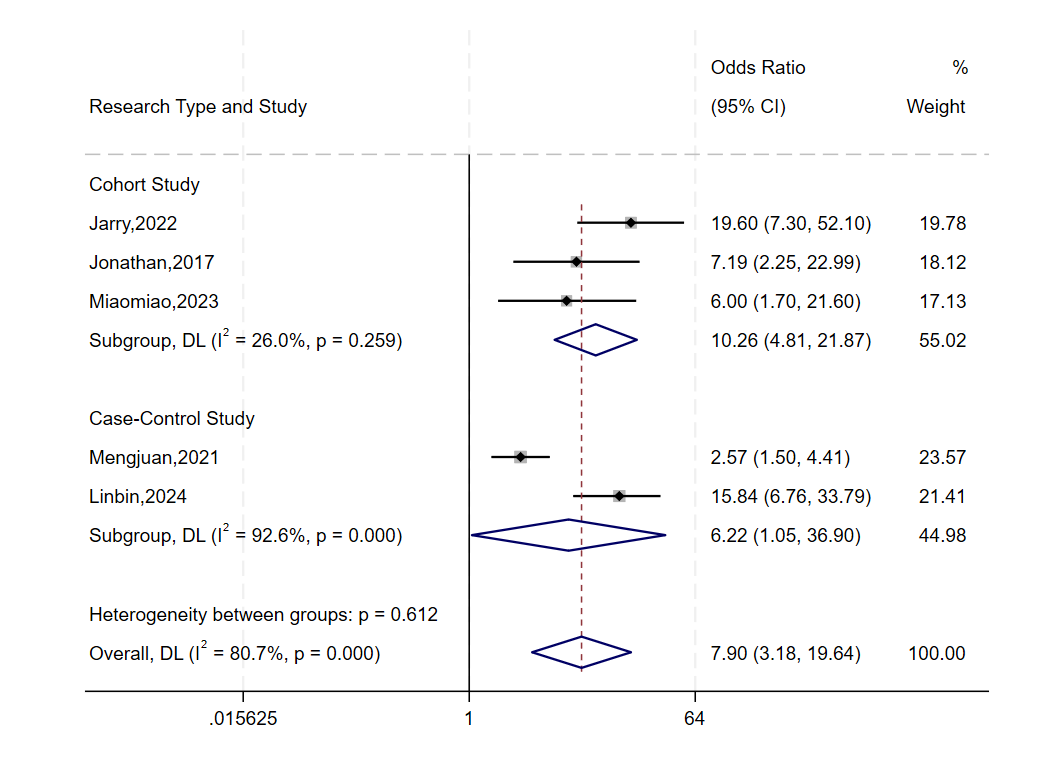
**

**Figure 5：History of urinary tract infection- Subgroup analysis -****Research Type**

**
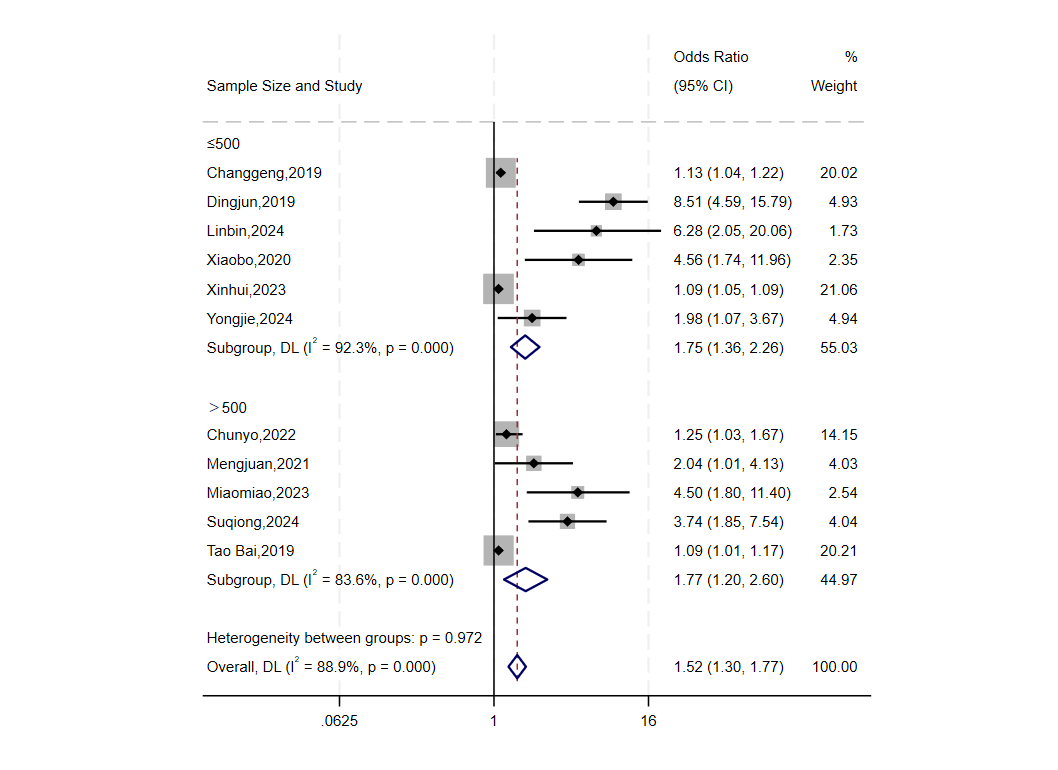
**

**Figure 6：Operation Time-Subgroup Analysis -Sample Size**

**
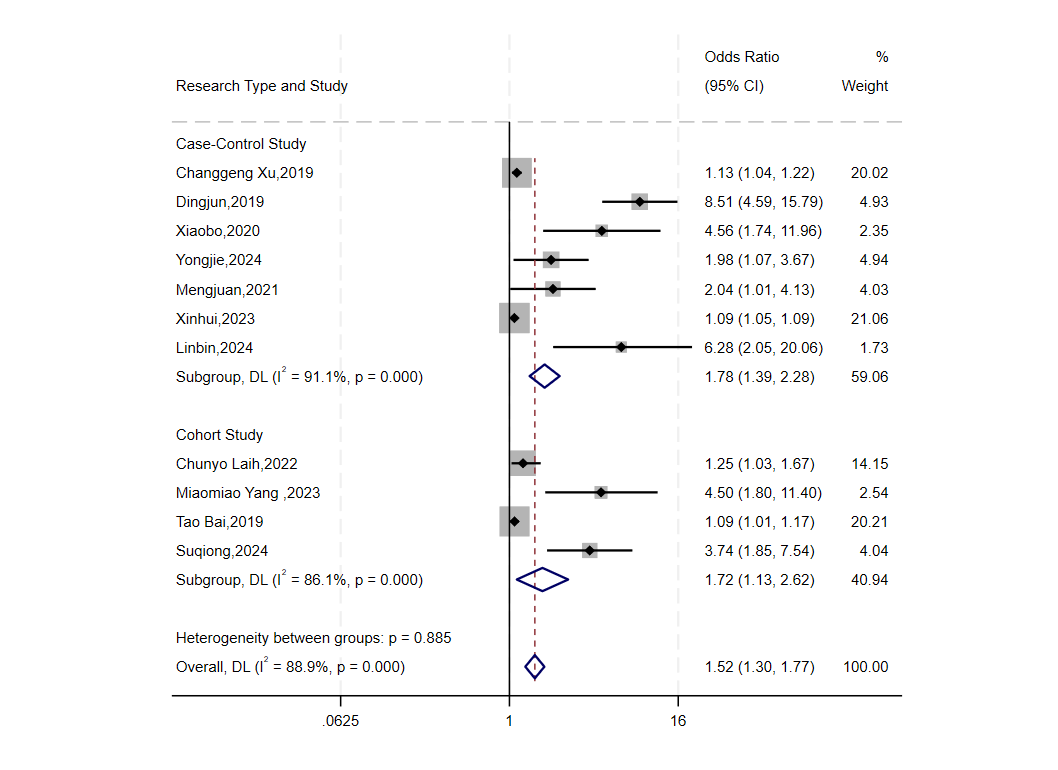
**

**Figure 7：Operation Time-Subgroup Analysis -Research Type**

**
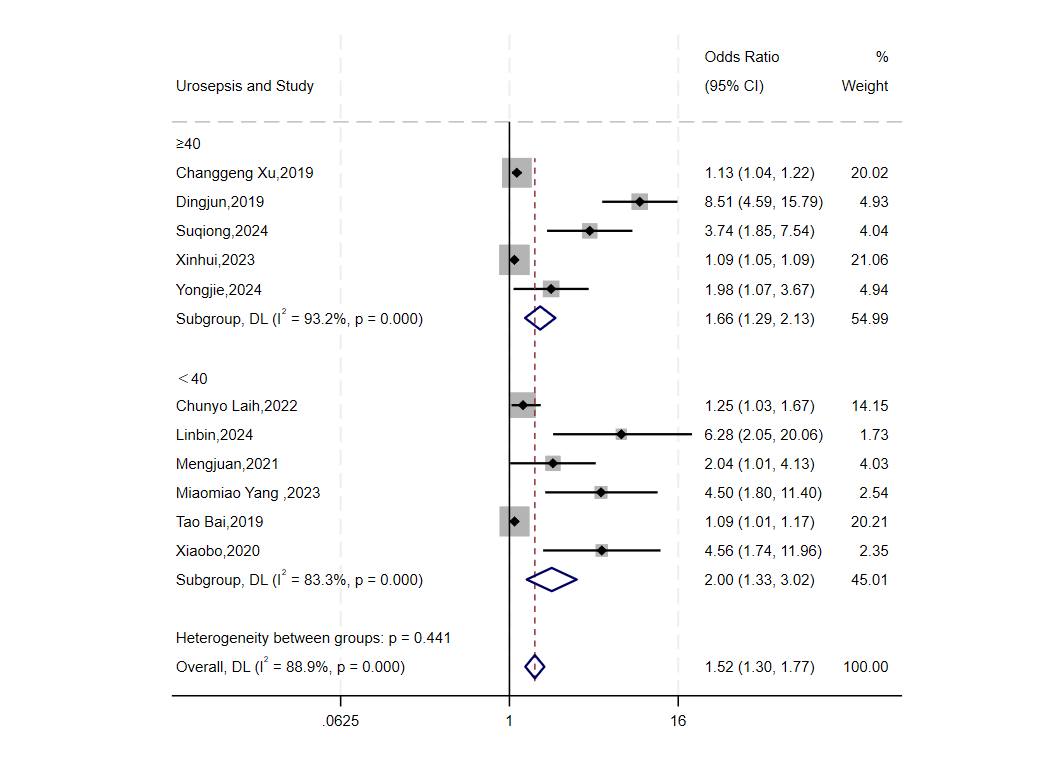
**

**Figure 8：Operation Time-Subgroup Analysis -Urosepsis**

**
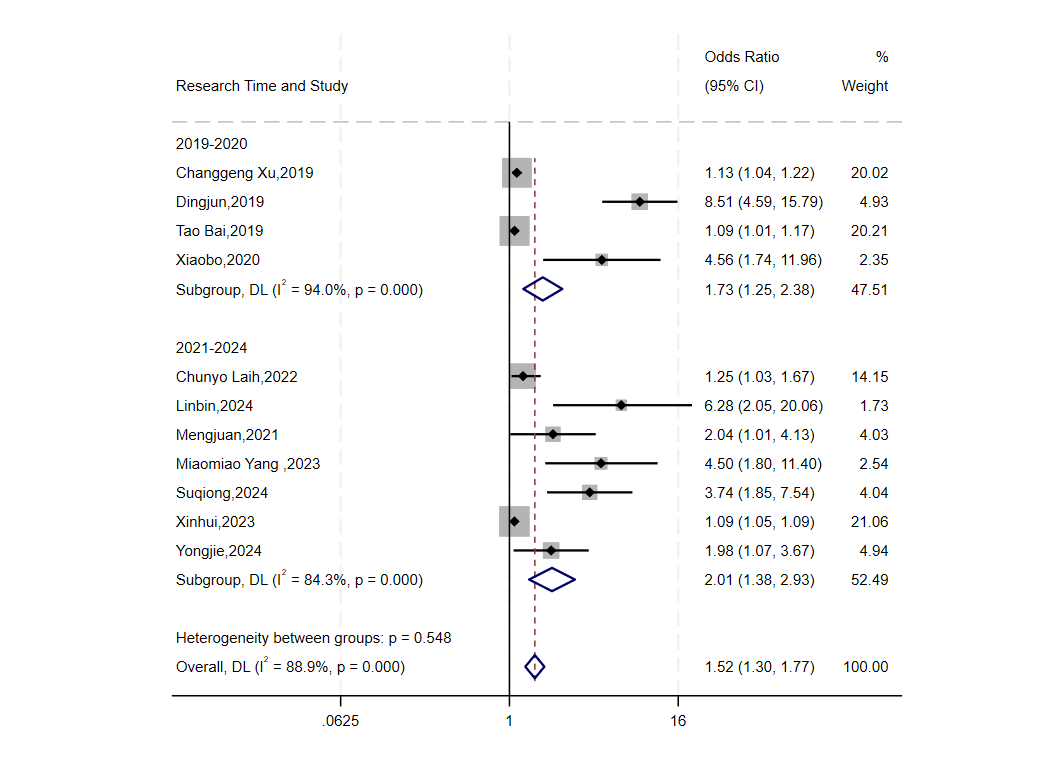
**

**Figure 9：Operation Time-Subgroup Analysis -Research Time**

**
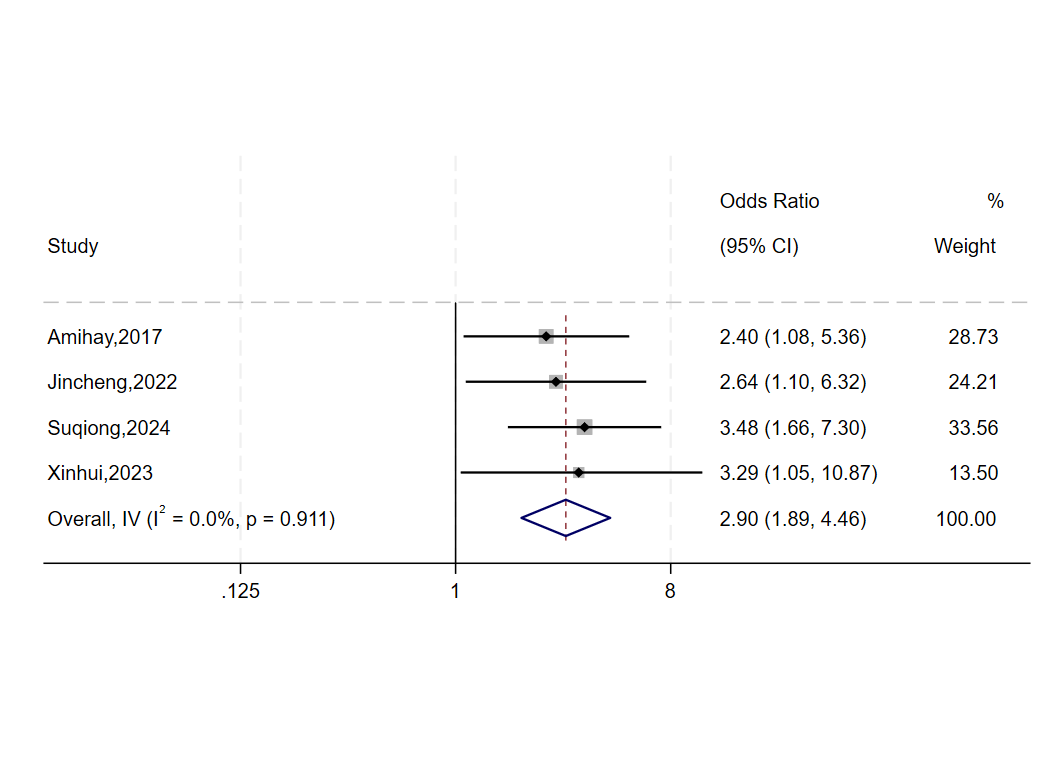
**

**Figure 10: Gender-Sensitivity Analysis**

**
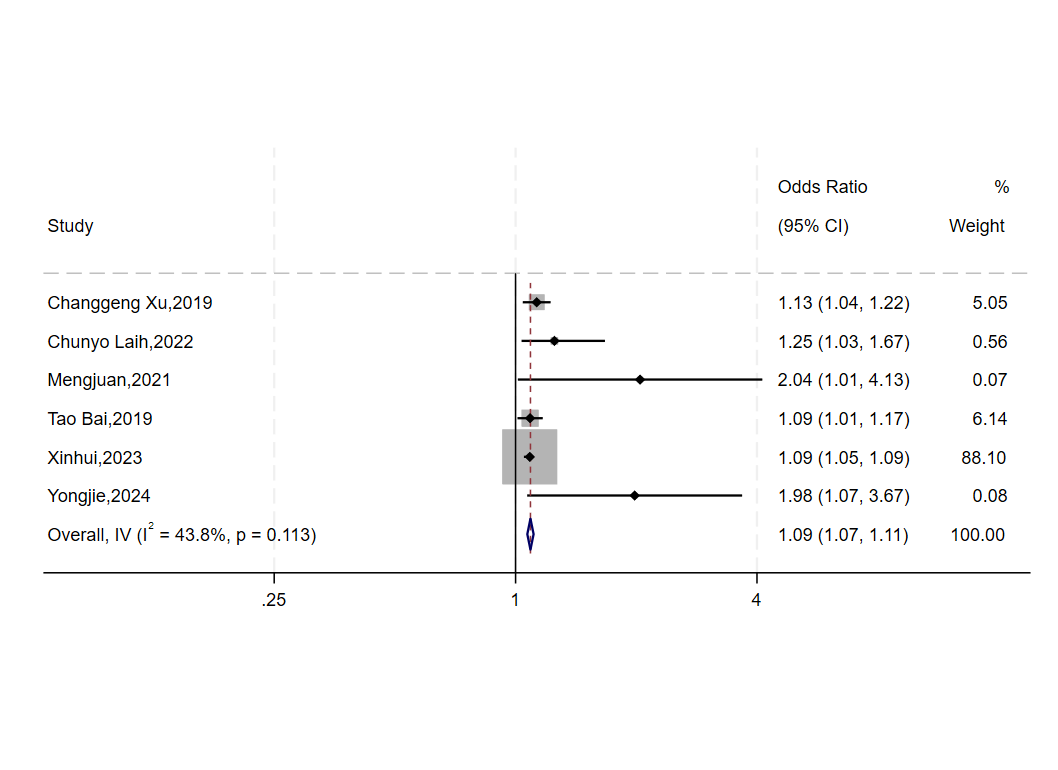
**

**Figure 10: Operation Time-Sensitivity Analysis**
