# Supplementary material for: Risk factors for urosepsis following ureteroscopic lithotripsy: a systematic review and meta-analysis
Source: Front Surg. 2025 Jun 19;12:1603311. doi: 10.3389/fsurg.2025.1603311 (PMC12222153; doi:10.3389/fsurg.2025.1603311)
Supplement: Supplementary file 2 [file Datasheet2.docx]

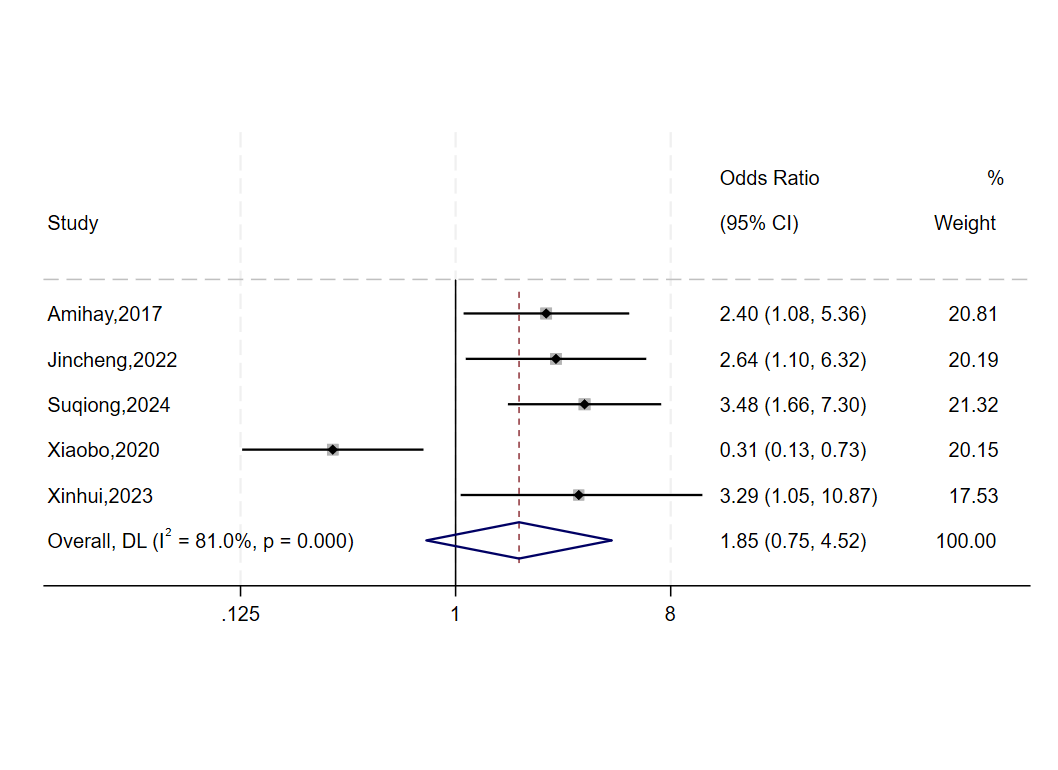
Figure 1：gender


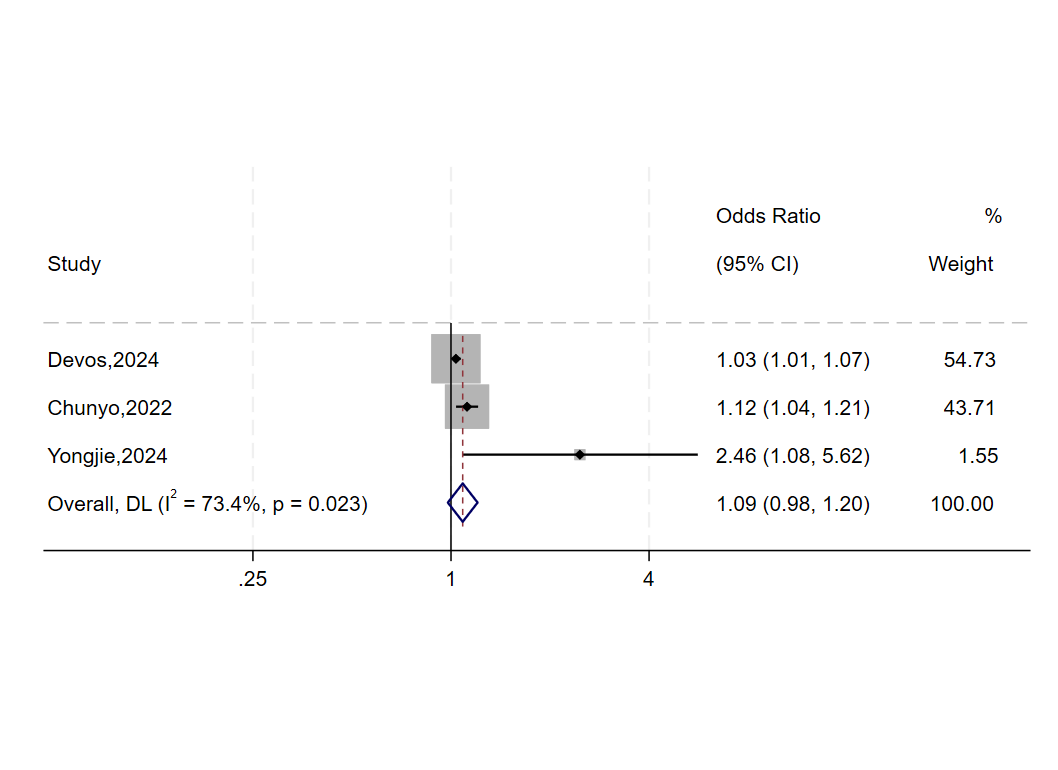
Figure 2：Age

**
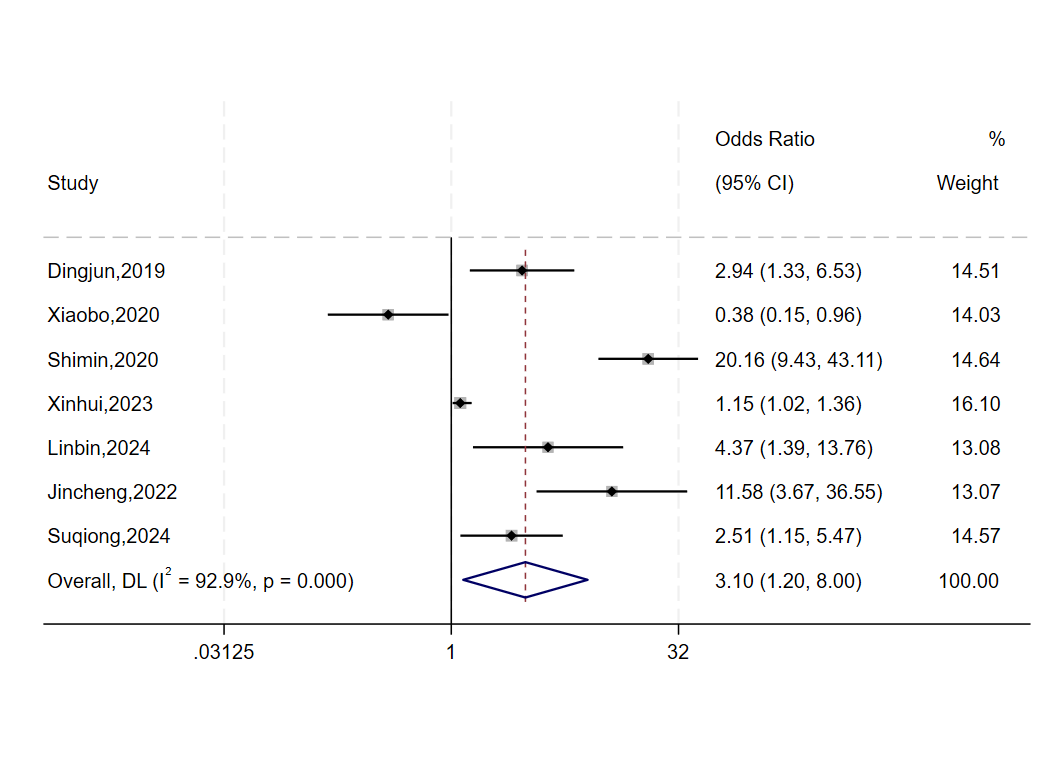
**Figure 3：Stone size

**
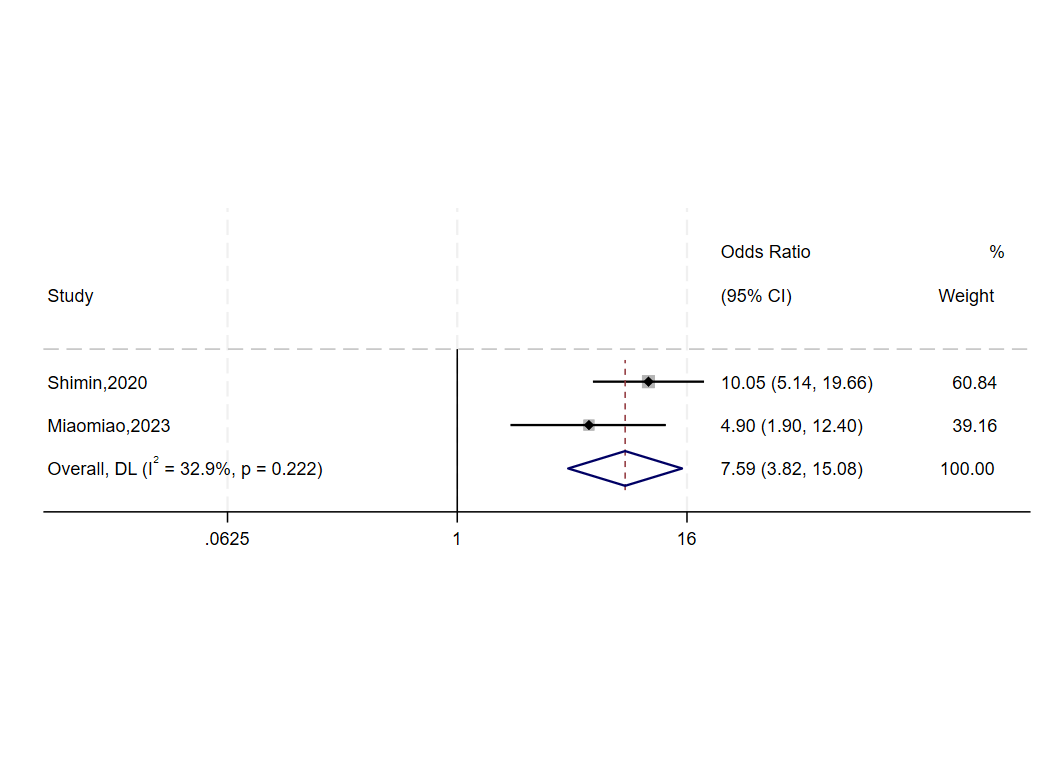
**

Figure 4：Number of stones


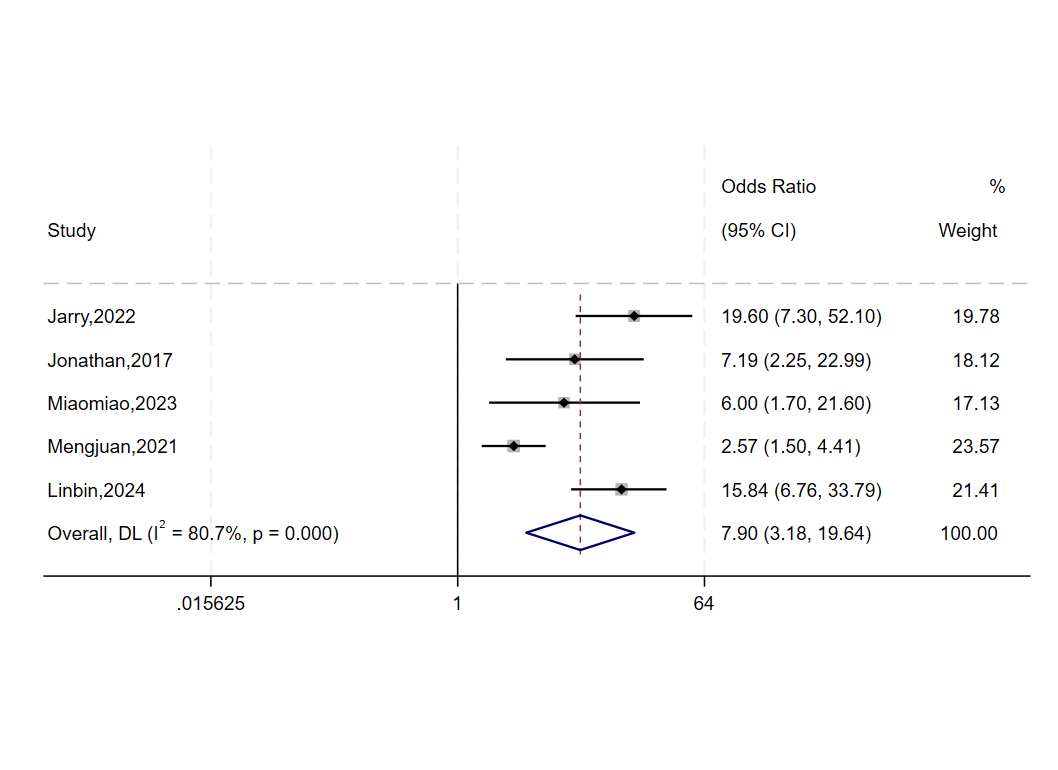


Figure 5：History of urinary tract infection

**
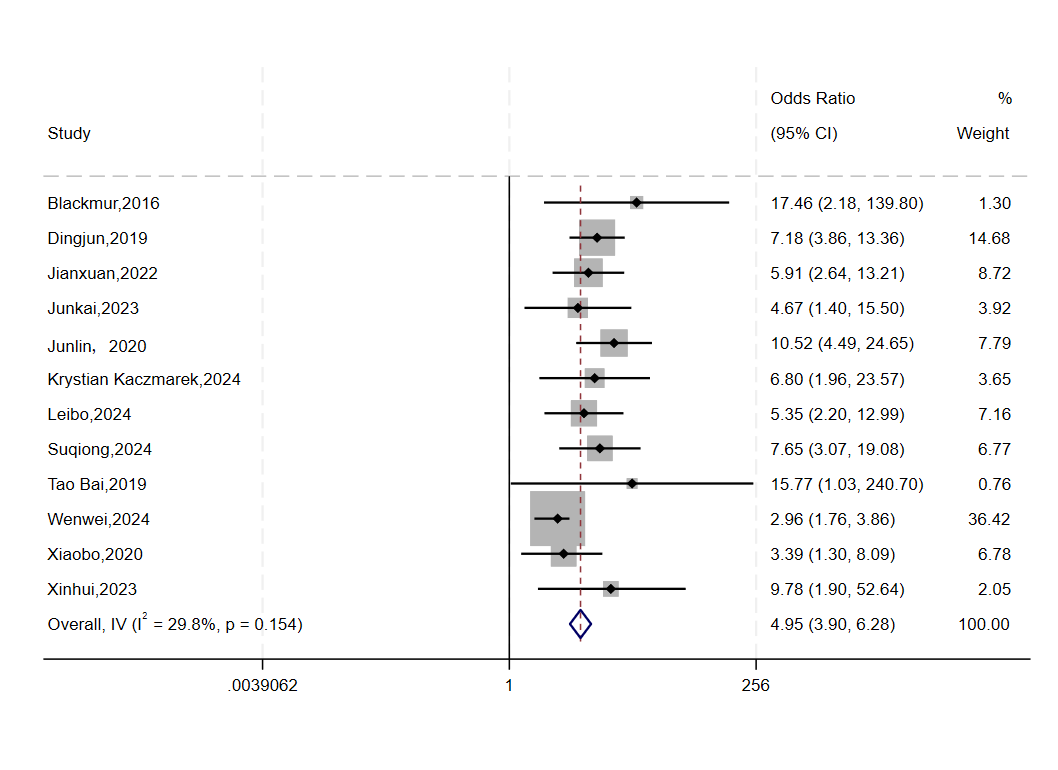
**

Figure 6：Positive urine culture

**
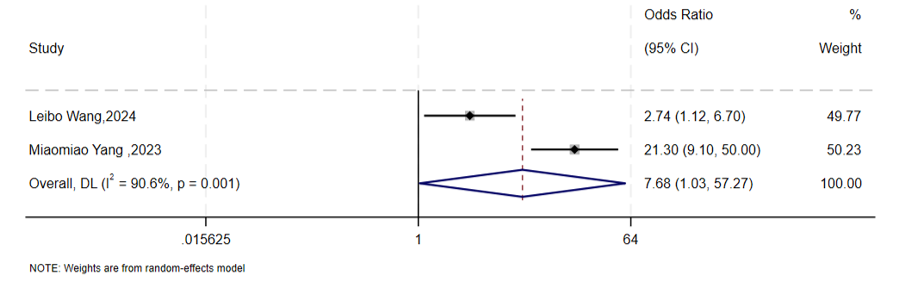
**

Figure 7：Nitrite in urine positive

**
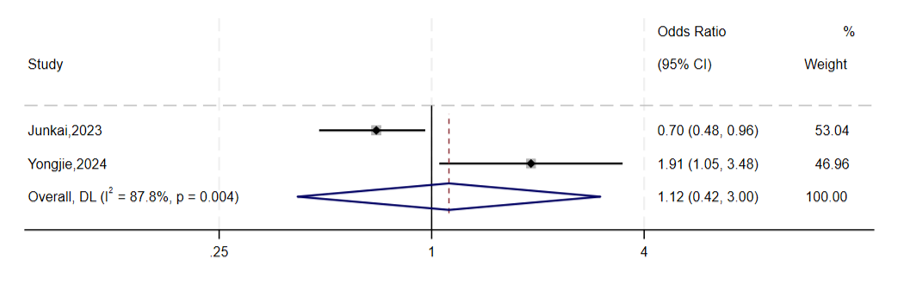
**

Figure 8：White blood cell count

**
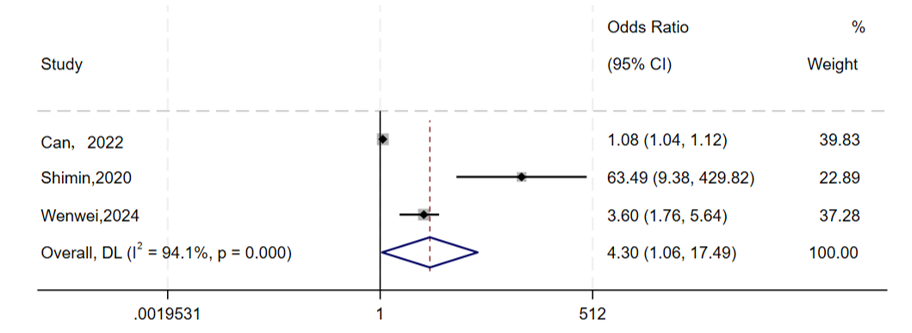
**

Figure 9：C-reactive Protein

**
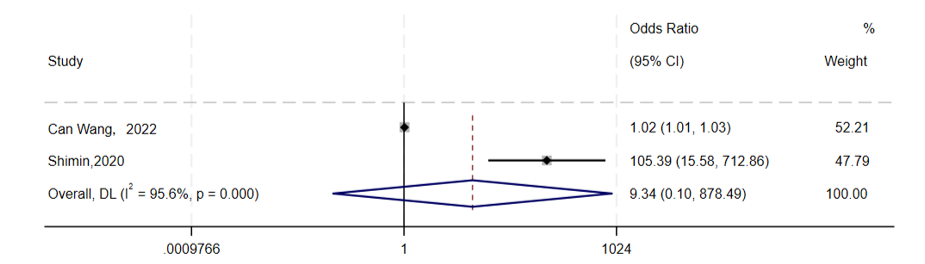
**

Figure 10：Procalcitonin

**
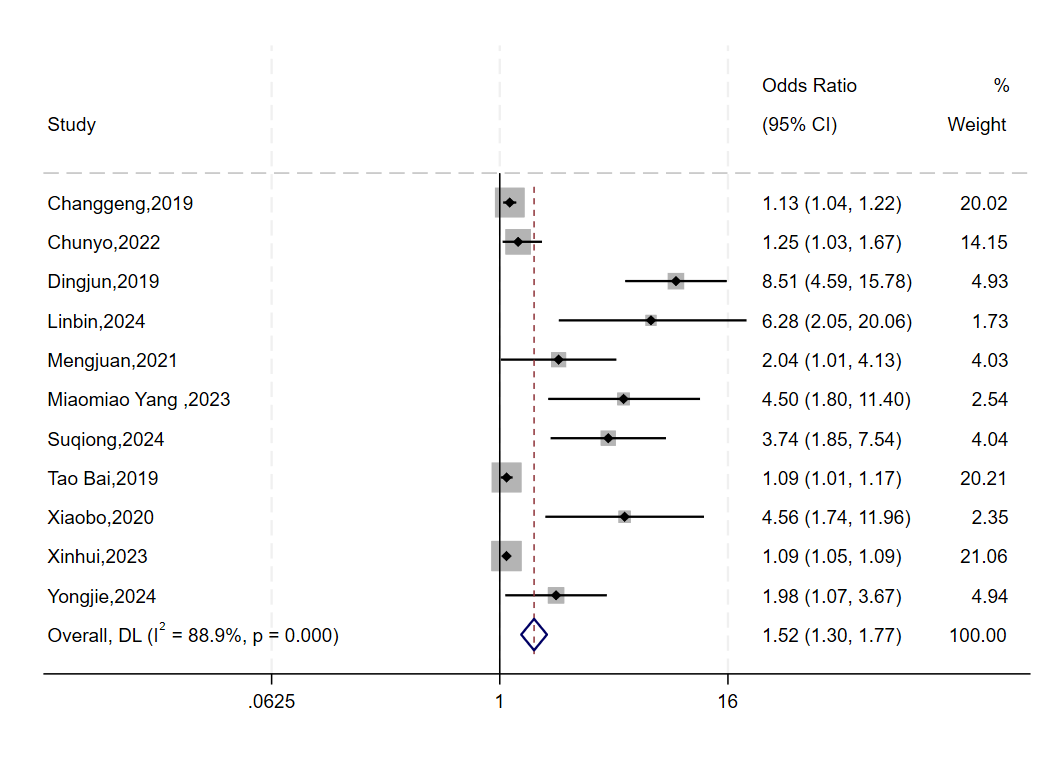
**Figure 11：Operation time

**
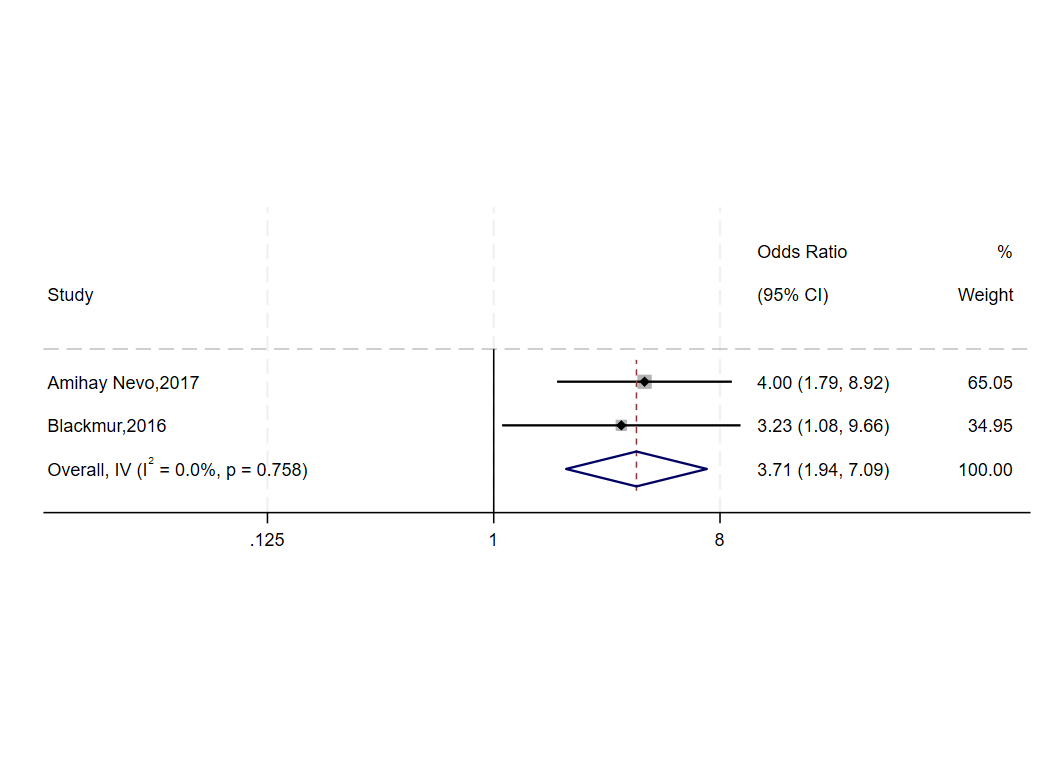
**

Figure 12：Stent placement

**
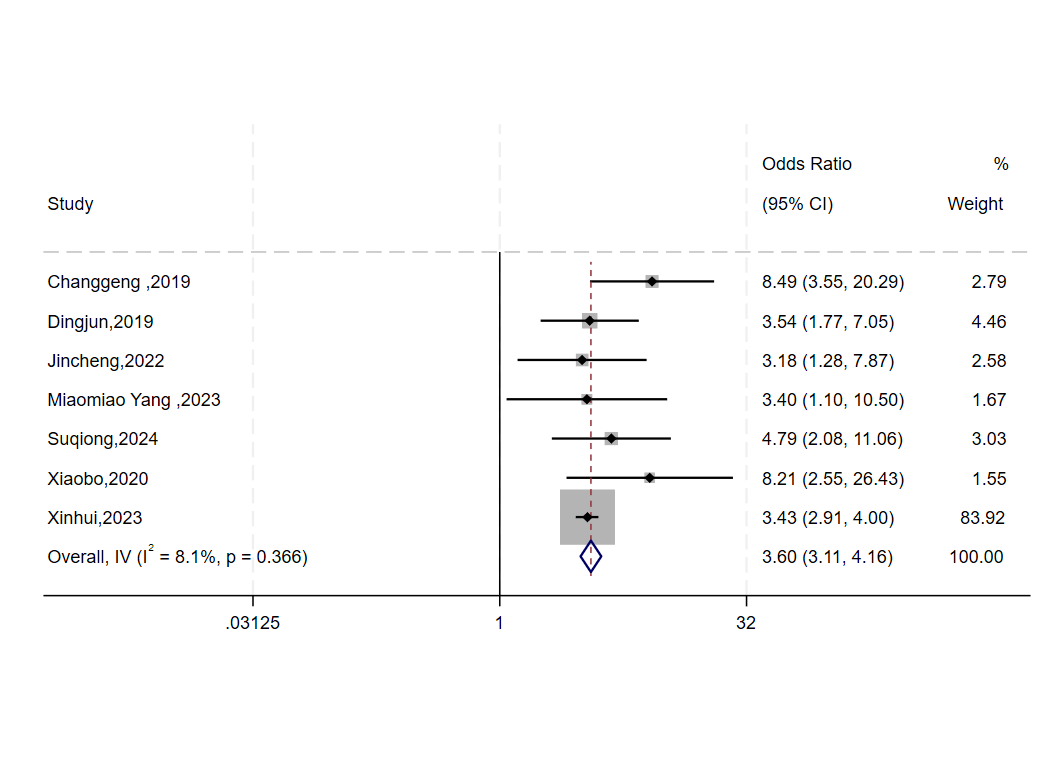
**Figure 13：Diabetes
